# Structural Modifications of Hybrid O-Alkylsulfonyl-β-(Benzimidazol-1-yl)propioamidoximes as a Pathway to the Antimicrobial, Antifungal and Antidiabetic Drugs

**DOI:** 10.3390/ijms27146224

**Published:** 2026-07-12

**Authors:** Lyudmila Kayukova, Anna Vologzhanina, Ekaterina Dubasova, Roza Seidakhmetova, Azamat Yerlanuly, Aruzhan Sartoyeva, Aigul Malmakova

**Affiliations:** 1 Laboratory of Chemistry of Synthetic and Natural Drug Substances, JSC A.B. Bekturov Institute of Chemical Sciences, 106 Shokan Ualikhanov Str., Almaty 050010, Kazakhstan; azaraze8575@mail.ru (A.Y.); aruzhansartaeva01@gmail.com (A.S.); malmakova@mail.ru (A.M.); 2 X-Ray Diffraction Laboratory, A.N. Nesmeyanov Institute of Organoelement Compounds, Russian Academy of Sciences, Moscow 119334, Russia; vologzhanina@mail.ru; 3 Laboratory of Quantum Chemistry, N.S. Kurnakov Institute of Inorganic and General Chemistry, Russian Academy of Sciences, Moscow 119991, Russia; dubasova.ek@gmail.com; 4 Department of Clinical Pharmacology and Evidence-Based Medicine, Karaganda Medical University, Karaganda 100012, Kazakhstan; roza77124@gmail.com

**Keywords:** benzimidazole core, an amidoxime group, O-alkylsulfonyl moiety, hybridization, X-ray diffraction, in vitro antimicrobial, antifungal and antidiabetic activities

## Abstract

The continuous search for new chemical structures more active and less toxic than those currently in practice is justified on the basis of the use of proven pharmacophoric building blocks (benzimidazole heterocycle, sulfonyl group and amidoxime framework in our case). The aim of the work was to synthesize new O-alkylsulfonyl-β-(benzimidazol-1-yl)propioamidoximes and to test them for in vitro biological activities to identify potentially effective agents. Novel O-alkylsulfonylamidoximes were synthesized in hydrochloride and base forms by the reaction of β-(benzimidazol-1-yl)propioamidoxime with alkylsulfonyl chlorides AlkSO_2_Cl (Alk = CH_3_, *n*-C_3_H_7_, *i*-C_3_H_7_, *n*-C_4_H_9_) in a mixture of water:acetone in hydrochloride and base forms. Hydrochlorides and bases of the O-alkylsulfonyl-β-(benzimidazol-1-yl)propioamidoximes were obtained in moderate to high yields. The structures of the synthesized compounds were established by physicochemical and spectral (elemental analysis, FT-IR, NMR and X-ray diffraction) methods. The obtained derivatives were tested for antimicrobial, antifungal and antidiabetic activity. Biological screening found effective samples of amidoximes with antimicrobial and antifungal activities that were near or exceeded the activity of the reference drugs gentamicin and nystatin; in addition, two samples with antidiabetic activity higher than acarbose were found. The results of the present study open new possibilities for the novel β-aminopropioamidoxime class as active antimicrobial and antifungal agents, as well as antidiabetic ones.

## 1. Introduction

### 1.1. Hybridization Direction in Medicinal Chemistry

The search for new drugs increasingly employs hybridization—the covalent combination of two or more pharmacophoric units into a single molecule [1]. This strategy addresses limitations of single-target therapies, such as drug resistance, and challenges of combination therapies, including unpredictable pharmacokinetics and toxicity [2]. Hybridization creates multi-target-directed ligands (MTDLs), which offer simplified dosing, improved pharmacokinetic profiles, and potential cost benefits [3]. Consequently, many MTDLs have entered clinical practice [4].

Our research focuses on synthesizing hybrid molecules like O-alkylsulfonyl-β-(benzimidazol-1-yl)propioamidoximes, comprising three established pharmacophores: amidoxime, alkylsulfonyl, and benzimidazole fragments.

### 1.2. The Strategic Value of the Amidoxime Moiety in Drug Development

Amidoximes exhibit a broad spectrum of activity against various microorganisms and complex eukaryotic organisms. As such, they are intensively studied as potential drugs, prodrugs, fungicides, or bactericides [5,6], and as exogenous sources of nitric oxide (NO) [7].

Amidoximes exhibit broad bioactivity and are studied as potential drugs, prodrugs, and antimicrobials. Their utility as prodrugs stems from good oral absorption and subsequent enzymatic conversion via the mitochondrial amidoxime reducing complex (mARC) to the active amidine form [8]. Depending on the reduction steps required for the transition to the amidine form, secondary and tertiary prodrugs are distinguished [9]. Drugs containing such prodrug groups accounted for over 13% of all FDA-approved small-molecule entities between 2012 and 2022 [10].

This amidine group is a fragment of L-arginine, a vital amino acid with numerous pharmacological roles [11]. While amidine-based drugs (e.g., pentamidine) show potent antiparasitic, antimicrobial, and anticancer activity [12,13,14], their poor oral bioavailability [15] and protonation at physiological pH values, creating a charged form, hinder the ability to cross non-polar cell membranes and make their clinical use difficult [16]. The amidoxime prodrug strategy effectively circumvents these limitations, improving pharmacokinetics without compromising activity [17].

### 1.3. Benzimidazole: A Privileged Scaffold in Pharmaceutical and Agrochemical Discovery

The benzimidazole moiety is a privileged structure with enormous therapeutic potential, primarily because its core structure closely resembles that of purine, a vital biological heterocycle. The ability of all living organisms—eukaryotes, bacteria, and archaea—to perform de novo purine biosynthesis underscores the fundamental role of purines in life [18]. Due to this similarity with natural nucleotides, benzimidazole derivatives can readily interact with diverse biomacromolecules and target proteins. This interaction underpins their wide spectrum of pharmacological activities, which includes antibacterial, antifungal, antidiabetic, anticancer, antiparasitic, analgesic, antiviral, and antihistamine effects. Consequently, they have found clinical application in treating cardiovascular diseases, neurological and endocrine disorders, and in ophthalmology [19].

Beyond human medicine, the utility of benzimidazole-based compounds extends to the agricultural sector, where they are effectively employed as fungicides and pesticides [20].

### 1.4. The Therapeutic and Agrochemical Impact of Sulfonyl-Containing Compounds

To improve the pharmacological properties of new drugs, sulfur-containing groups are included as an essential element in drug structures. This is evidenced by reviews of FDA-approved drugs up to 2018, 2019, and for the period 2020–2024. Organosulfur compounds constitute almost 25% of all small-molecule medications in use from 2017 to 2020 [21,22].

Important classes of agents extensively used as both pharmaceuticals and agrochemicals are those featuring sulfonyl or sulfonamide functional groups [23,24]. Sulfonamides, originating from the antibacterial sulfanilamide, now include agents like acetazolamide, glibenclamide, and anticancer compounds [25,26]. Sulfonylureas are cornerstone treatments for type 2 diabetes [27].

In agrochemistry, organosulfur compounds are vital, with sulfur present in over 30% of modern pesticides, chiefly fungicides, herbicides, and insecticides. Sulfonylureas and sulfonamides are among the most prominent classes [28]. Key sulfonylurea herbicides, such as bensulfuron-methyl, nicosulfuron, and thifensulfuron-methyl, are applied widely to cereals, fruits, and vegetables [29,30]. The herbicidal action of both sulfonylureas and sulfonamides is based on the inhibition of the enzyme acetolactate synthase (ALS). This enzyme is essential for the biosynthesis of branched-chain amino acids; its inhibition disrupts weed growth [31].

*The motivation for this work* lies in the development of the sulfochlorination chemistry of β-aminopropioamidoximes, revealing a key mechanistic aspect of alkylsulfochlorination and its differences from arylsulfochlorination. Furthermore, establishing the fine structural features of the products using modern spectral methods, the main one being X-ray crystallography, was of interest. Combining pharmacophoric building blocks in the novel hybrid O-alkylsulfonyl-β-(benzimidazol-1-yl)propioamidoximes and their in vitro antimicrobial, antifungal, and antidiabetic screening suggested the discovery of promising drug candidates. *The objectives of this work* were: (1) to synthesize novel alkylsulfonyl derivatives of β-(benzimidazol-1-yl)propioamidoxime by combining known pharmacophoric fragments; (2) to unequivocally characterize the structures of the resulting sulfochlorination products using a comprehensive suite of physicochemical methods, including FT-IR, ^1^H/^13^C NMR, and X-ray crystallography; and (3) to evaluate their potential as drug candidates through in vitro screening, assessing activity against common bacterial and fungal pathogens, as well as for relevance to socially significant diseases such as diabetes mellitus.

## 2. Results and Discussion

### 2.1. Synthesis of O-Alkylsulfonyl-β-(Benzimidazole-1-yl)propioamidoximes in a Water:Acetone Mixture Without Base

β-(Benzimidazole-1-yl)propioamidoxime (**1**) is not a commercially available compound. Its ‘one put’ two-step synthesis and characterization are part of Section 3. The synthesis of compound (**1**) involves the reaction of benzimidazole with acrylonitrile to form β-(benzimidazole-1-yl)propionitrile without isolation; the reaction of the latter with hydroxylamine base yields β-(benzimidazole-1-yl)propioamidoxime (**1**) in 56% yield.

**Scheme 1:**
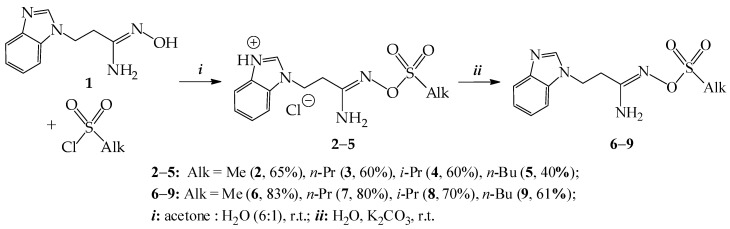
Alkylsulfochlorination of β-(benzimidazol-1-yl)propioamidoxime (**1**) in the absence of a base.

Given the presence of internal basic centers in the β-(benzimidazol-1-yl)propioamidoxime (**1**), its alkylsulfochlorination was performed without an external base, utilizing its internal benzimidazole nitrogen to bind the released HCl, as confirmed by X-ray analysis. This base-free reaction in a water/acetone mixture at r.t. produced hydrochlorides **2**–**5** in 40–83% yields [Scheme 1(***i***), Table 1].

Hydrochloride **2** is caramel-like masses for which it is impossible to determine the melting point; at the same time O-*n*-propylsulfonyl-, O-*i*-propylsulfonyl- and O-*n*-butylsulfonyl-β-(benzimidazol-1-yl)propioamidoxime hydrochlorides (**3**–**5**) were isolated as crystalline substances with melting points of 89–91, 163–165 and 82–84 °C. Hydrochlorides **2**–**5** were converted into bases **6**–**9** by the action of aqueous potash [Scheme 1(***ii***), Table 1].

**Figure 1:**
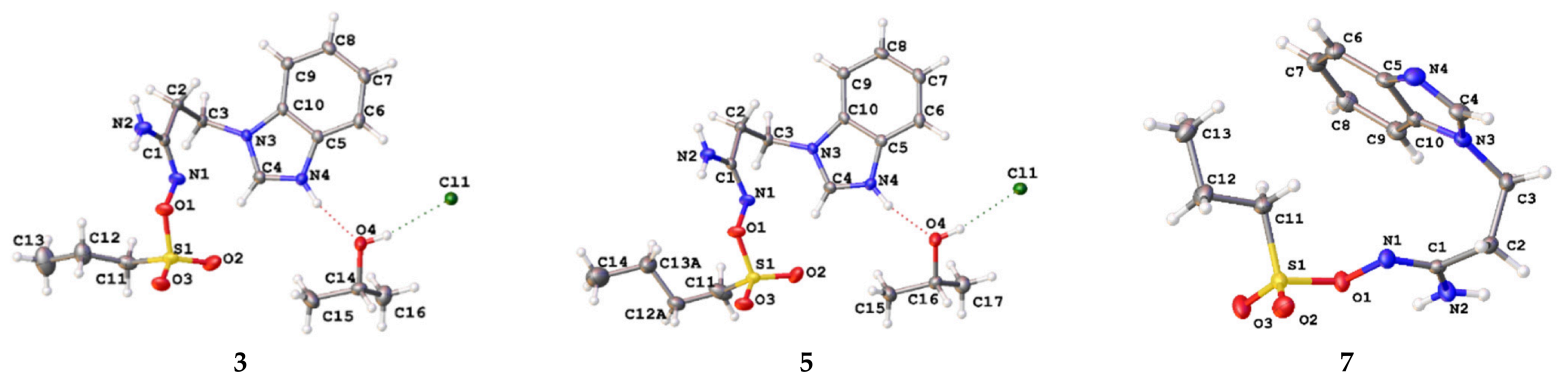
Asymmetric units of hydrochlorides **3**, **5** and base **7** of O-alkylsulfonyl-β-(benzimidazole-1-yl)propioamidoximes. The structures of molecules **3** and **5** contain an isopropyl alcohol molecule.

**Figure 2:**
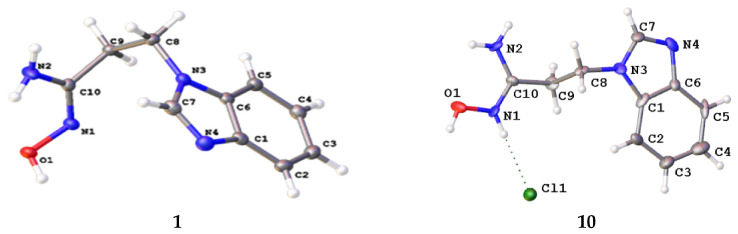
Molecular view of β-(benzimidazol-1-yl)propioamidoxime (**1**), β-(benzimidazol-1-yl)propioamidoxime hydrochloride (**10**) isolated under conditions (***iii***).

Compounds **1**–**9** were characterized by physicochemical (Table 1) and spectral data (FT-IR spectroscopy, ^1^H- and ^13^C-NMR spectroscopy) (see Supplementary Data, pages S1–S32; X-ray diffraction data (Figure 1 and Figure 2; Tables S1 and S2).

The most characteristic FT-IR bands and the most representative ^1^H- and ^13^C-NMR signals for alkylsulfochlorination products **2**–**9** are described below.

In the FT-IR spectra, the most convincing evidence for the formation of compounds **2**–**9** was the presence of asymmetric and symmetric S=O stretching vibrations in the regions of 1347–1361 cm^−1^ and 1160–1194 cm^−1^, respectively. Furthermore, the spectra of hydrochlorides **2**–**5** exhibited a broad band in the 2700 cm^−1^ region, which is characteristic of N(+)–H stretching vibrations.

The ^1^H-NMR spectra of hydrochlorides **2**–**5** displayed signals (δ, ppm) for the alkylsulfonyl group protons as follows: **2**: 2.46 (s, 3H, CH_3_); **3**: 0.82 (t, J = 7.5 Hz, 3H, CH_2_CH_2_CH_3_), 1.47 (m, 2H, CH_2_CH_2_CH_3_), 2.90 (t, J = 7.5 Hz, 2H, CH_2_CH_2_CH_3_); **4**: 1.01 (d, J = 7.0 Hz, 6H, CH(CH_3_)_2_), 3.11 (m, 1H, CH(CH_3_)_2_); **5**: 0.77 (t, J = 7.0 Hz, 3H, CH_2_CH_2_CH_2_CH_3_), 1.22 (m, 2H, CH_2_CH_2_CH_2_CH_3_), 1.43 (m, 2H, CH_2_CH_2_CH_2_CH_3_), 2.98 (t, J = 7.0 Hz, 2H, CH_2_CH_2_CH_2_CH_3_).

In the ^13^C-NMR spectra the signals (δ, ppm) for the alkylsulfonyl carbon atoms of hydrochlorides **2**–**5** were observed at: **2**: 18.2 (CH_3_); **3**: 16.3, 31.31, 49.0 (*n*-Pr); **4**: 16.2, 48.9 (*i*-Pr); **5**: 13.9, 21.1, 25.3, 47.6 (*n*-Bu). For the corresponding free bases **6**–**9**, the signals for these alkylsulfonyl groups in both the ^1^H- and ^13^C-NMR spectra, as a rule, were consistently shifted upfield compared to their hydrochloride salts.

Single crystals of all O-alkylsulfonyl-β-(benzimidazol-1-yl)propioamidoximes (**2**–**9**), grown by recrystallization from isopropyl alcohol, were subjected to X-ray diffraction analysis. However, only the crystals of compounds **3**, **5** and **7** were of sufficient quality for data collection (Figure 1, Table S1).

All the synthesized compounds are novel, and their structures were previously uncharacterized. Compounds **3** and **5** are isostructural, differing solely in the nature of the alkyl chain.

Their crystal structures each comprise a cation (protonated at the heterocyclic nitrogen atom), a chloride counterion, and a molecule of isopropyl alcohol from the recrystallization solvent. A comparison of the solid-state molecular structures of **3** and **7** reveals significant conformational flexibility, which is manifested in their extended and folded conformations, respectively.

Thus, the presence of an internal basic center—the Nsp^2^ atom of the imidazole heterocycle in the β-(benzimidazol-1-yl)propioamidoxime molecule—enables its alkylsulfochlorination in a water-acetone solvent system without requiring an external base. This approach successfully afforded a series of O-alkylsulfonyl-β-(benzimidazol-1-yl)propioamidoxime hydrochlorides (**2**–**5**). These salts were subsequently converted into the corresponding free bases (**6**–**9**) by treatment with aqueous potassium carbonate (Table 1).

### 2.2. Reaction of β-(Benzimidazole-1-yl)propioamidoxime with Alkylsulfonyl Chlorides in CHCl_3_ in the Presence of Bu_3_N (**iii**)

**Scheme 2:**
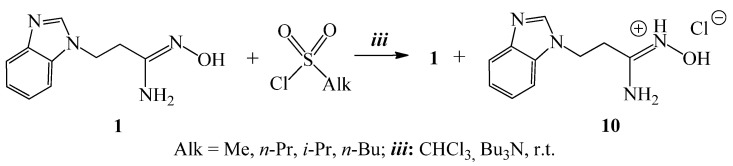
An attempt of β-(benzimidazol-1-yl)propioamidoxime (**1**) alkylsulfochlorination in the presence of a base resulted in the unexpected formation of β-(benzimidazol-1-yl)propioamidoxime hydrochloride (**10**).

To obtain the target O-alkylsulfonylation products of β-(benzimidazol-1-yl)propioamidoxime (**1**), we also used a standard base-mediated (chloroform, Bu_3_N) alkylsulfochlorination strategy. This approach considers the oxygen atom of the amidoxime group as ‘a priori’ the most likely nucleophilic center. The reactions were performed in chloroform using Bu_3_N as a base to scavenge the released HCl (Scheme 2, conditions ***iii***). A series of alkylsulfonyl chlorides (AlkSO_2_Cl) were used, where Alk = Me, *n*-Pr, *i*-Pr, *n*-Bu.

This strategy was chosen based on our prior success in synthesizing analogous O-arylsulfonyl derivatives from compound **1** using arylsulfonyl chlorides (XC_6_H_4_SO_2_Cl, where X = *p*-CH_3_, *o*-NO_2_, *p*-NO_2_) in chloroform with DIPEA as a base [32,33].

In the present study, DIPEA was not tested as a base, although it can be assumed that, due to its steric hindrance and hence lower nucleophilicity compared to Bu_3_N, DIPEA would not enter into a competing side reaction for binding the alkyl sulfonyl chloride. Bu_3_N was chosen to test the effect of the base in the alkylsulfochlorination reaction based on economic considerations, as it is 10 times cheaper than DIPEA. As shown in the previous section of our study, Section 3.1, no external base is required at all to obtain the O-alkyl sulfochlorination products of β-(benzimidazole-1-yl)propioamidoxime.

Upon completion of the reactions under conditions (***iii***), as monitored by TLC, the crystalline products isolated in approximately 50% yield were not the anticipated O-alkylsulfonyl derivatives. Comprehensive analysis by FT-IR, ^1^H- and ^13^C-NMR spectroscopy, and X-ray crystallography revealed that the isolated compounds were, instead, the starting amidoxime **1** and its hydrochloride salt **10** (Scheme 2, Table 1, Figure 2). A representative procedure for the reaction of **1** with isopropylsulfonyl chloride in chloroform in the presence of Bu_3_N is provided in Section 3.

X-ray structural data of amidoxime **1** are presented here for the first time. Similarly, a comprehensive physicochemical and spectral characterization (including FT-IR, ^1^H-NMR, ^13^C-NMR, and X-ray diffraction) of hydrochloride **10** is also reported for the first time.

The FT-IR spectrum of amidoxime **1**, as a distinctive feature of the unsubstituted –NOH group, shows a stretching vibration band for the O–H bond at ν = 3420 cm^–1^. In contrast, the spectrum of hydrochloride **10** shows a band at ν = 2300 cm^−1^, characteristic of the N(+)–H ammonium bond, confirming its formation.

Furthermore, the ^1^H-NMR spectra of both compound **1** and hydrochloride **10** contain signals for all functional groups. As expected, all the proton signals in hydrochloride **10** are shifted downfield compared to those of the original amidoxime **1**. The ^13^C-NMR spectra revealed a consistent trend: all the carbon atom signals of amidoxime **1** appeared at higher fields compared to those of its hydrochloride salt **10**.

Most significantly, the X-ray structural data establish that protonation occurs specifically at the nitrogen atom of the oxime group (Figure 2, Table S2).

**Scheme 3:**
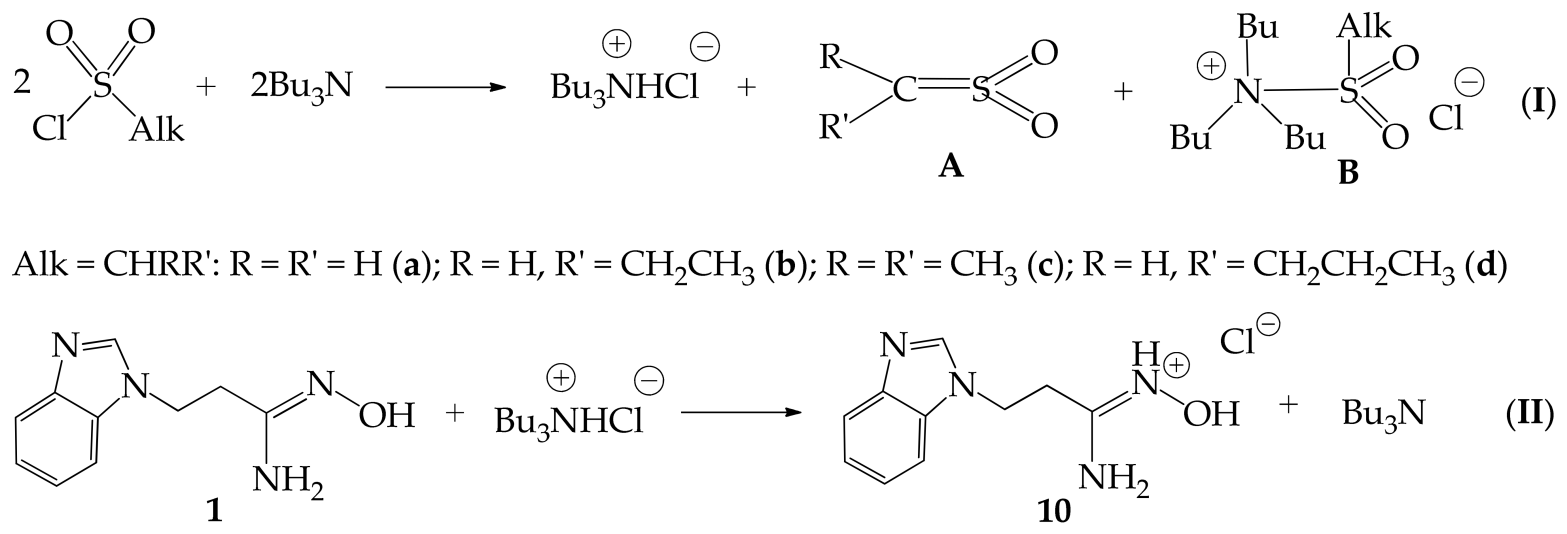
(Addition to Scheme 2). Plausible ways for the removal of alkyl sulfonyl chlorides from the reaction with β-(benzimidazol-1-yl)propioamidoxime (**1**) under conditions ***ii*** in the presence of tributylamine with the formation of sulfenes **A** and the binding products of alkyl sulfonyl chlorides with tributylamine–alkylsulfonyltributylammonium chlorides **B** (**I**); formation of β-(benzimidazol-1-yl)propioamidoxime hydrochloride (**10**) (**II**).

A plausible mechanism for the formation of the observed products during the attempted alkylsulfochlorination of amidoxime **1** in the presence of Bu_3_N involves the disproportionation of alkylsulfonyl chlorides (Scheme 3, **I**). This reaction, which consumes two molecules of the sulfonyl chloride and two molecules of tributylamine, primarily yields tributylamine hydrochloride.

It is known that alkylsulfonyl chlorides possessing at least one α-hydrogen atom can react via a mechanism involving the highly reactive sulfene intermediate **A**. This sulfene species can then participate in subsequent transformations, leading to zwitterionic, episulfone, and stilbene products [34,35]. Subsequently, a competing reaction occurs where Bu_3_N, instead of the nucleophilic centers of amidoxime **1**, binds the alkylsulfonylchlorides to form alkylsulfonyltributylammonium chlorides (**B**). As a result, no sulfochlorination products of **1** were detected. Instead, hydrochloride **10** is formed via reaction between β-(benzimidazol-1-yl)propioamidoxime (**1**) and the tributylamine hydrochloride generated in the preceding step (**I**) (Scheme 3, **II**).

### 2.3. In Vitro Screening of O-Alkylsulfonyl-β-(Benzimidazole-1-yl)propioamidoximes (***2***–***9***) for Antimicrobial, Antifungal and α-Glucosidase Antidiabetic Activity

Compounds **2**–**9** were screened for antimicrobial, antifungal, and α-glucosidase inhibitory activity; the results are presented in Table 2.

*Antimicrobial and Antifungal Activity:* Five of the eight tested compounds demonstrated pronounced antimicrobial activity (12.5–50 µg/mL) against *S. aureus*: hydrochlorides **4** (Alk = *i*-Pr) and **5** (Alk = *n*-Bu) and free bases **7** (Alk = *n*-Pr), **8** (Alk = *i*-Pr), and **9** (Alk = *n*-Bu), with **5** and **8** (MIC = 12.5 µg/mL) being most potent.

Activity was also noted for hydrochlorides **2** (Alk =Me) and **5** (Alk = *n*-Bu) and free base **6** (Alk = Me) against *B. subtilis* with MIC 25–50 µg/mL and for hydrochlorides **2** (Alk = Me) and **3** (Alk = *n*-Pr) and free bases **6** (Alk = Me) and **7** (Alk = *n*-Pr) with MIC 12.5–25 µg/mL against *E. coli*. In contrast, none of the tested compounds showed activity against *P. aeruginosa*.

The synthesized O-alkylsulfonyl derivatives (**2**–**9**) exhibited more pronounced in vitro antifungal activity against *C. albicans* than antibacterial activity. Six of the eight tested compounds showed notable efficacy, with MICs ranging from 6.3 to 50 μg/mL. Free base **8** (Alk = *i*-Pr, MIC = 6.3 µg/mL) was twice as active as nystatin, while its hydrochloride counterpart **4** (Alk = *i*-Pr, MIC = 12.5 µg/mL) matched the standard. Compound **8** became the subject of the KZ Patent for Utility Model [36].

*Antidiabetic (α-Glucosidase) Activity:* Hydrochlorides **2** (Alk = Me) and **5** (Alk = *n*-Bu) exhibited significant inhibition (64.7% and 79.5%, respectively), exceeding the activity of acarbose (44.7%). These facts served as the basis for their protection by KZ Patents for Utility Model [37,38].

*Structure-Activity Relationship (SAR) analysis* should be performed taking into account (1) the hybrid structure of O-alkylsulfonyl-β-aminopropioamidoximes, (2) their chemical form (hydrochlorides or free bases), and (3) the fine structure of the alkyl group R in the O-alkylsulfonyl moiety.

*Hybrid structure*:

The molecular framework integrates three distinct bioactive motifs. Firstly, the amidoxime functional group, which contributes to biological efficacy through its ability to chelate metal ions critical for microbial enzyme function, as documented in previous studies [5,6,8].

Secondly, this intrinsic activity is complemented by the benzimidazole core as a well-established privileged structure in pharmaceuticals and agrochemistry, known for its potent and broad-spectrum antimicrobial and antifungal activities [19,20,21]. The synergy between these two fragments creates a robust foundation for the observed bioactivity.

Furthermore, the presence of the O-alkylsulfonyl moiety significantly augments this activity profile. The inclusion of a sulfur atom, particularly within a sulfonyl group, is a strategic design element. This is strongly supported by the fact that 25% of pharmaceuticals with sulfonyl or sulfonamide functional groups are known [22,23], the sulfonylurea-like moiety carrying antidiabetic potential [27], and over 30% of modern agrochemicals (including fungicides, herbicides, and insecticides) contain at least one sulfur atom [29,30,31].

*Chemical form (hydrochlorides or free bases)*:

Hydrochlorides **2**–**5** have better solubility in the aqueous test medium than bases **6**–**9**. However, this factor turned out not to confer any advantage in either antimicrobial or antifungal activity to the **2**–**5** hydrochloride group. Both groups (hydrochlorides **2**–**5** and bases **6**–**9**) have samples with pronounced activity. It has been shown that sometimes the superior solubility of hydrochlorides compared to bases interferes with target binding, as in the case of *C. albicans*, where free base **8** better penetrates membranes and exhibits superactivity.

α-Glucosidase is a rare case where only hydrochlorides **2** and **5** are active, likely due to the requirement for a positive charge for binding in the enzyme’s active site.The enzyme’s active site contains two critical negatively charged carboxylate residues—aspartate and glutamate—that act as the catalytic nucleophile and acid/base catalyst, respectively [39]. The protonated benzimidazole moiety of hydrochlorides **2** and **5** carries a positive charge, which can form a strong electrostatic (ion-pair) interaction with these anionic catalytic residues. This ionic anchor is a major contributor to binding affinity. In contrast, the neutral free bases (**6**–**9**) lack this positive charge and therefore cannot engage in this essential interaction, resulting in complete loss of inhibitory activity [40].

*Fine structure of the alkyl group R:*

- Me (compounds **2** and **6**) are active against *E. coli*, *B. subtilis*, and α-glucosidase, but not against *S. aureus* and weakly against *C. albicans*.
- *n***-**Pr (**7**) is active against *E. coli* and *S. aureus* (base) and *C. albicans* (base), but not against α-glucosidase.
- *i***-**Pr (**4** and **8** are the “stars” of antifungal activity in the hydrochloride form (**4**) and, especially, in the base form (**8**), and also perform well in antimicrobial tests—compounds **4** and **8** are active against *S. aureus*, but not against *E. coli* and α-glucosidase.
- *n***-**Bu is best for α-glucosidase (**5**), good against *S. aureus*, but not against *E. coli*.
- All compounds are inactive against *P. aeruginosa*, which is typical for many cationic and amphiphilic compounds due to the efficient efflux system and low permeability of the outer membrane [41].

Regarding the influence of the length and branching of the alkyl substituent of compounds **2**–**9** on α-glucosidase activity, it is known that the alkyl substituent governs hydrophobic interactions within a lipophilic subpocket of α-glucosidase [42]. Alkylsulfonyl derivatives show α-glucosidase inhibition that is highly dependent on alkyl chain length and branching [43]. While the small methyl group in **2** is accommodated, the *n*-propyl chain in **3** appears to be of insufficient length to reach a secondary hydrophobic binding domain, resulting in loss of activity. In contrast, the *n*-butyl chain in **5** reaches this distal lipophilic pocket, providing additional van der Waals contacts that significantly enhance binding affinity and inhibitory potency (79.5%). The lack of activity for isopropyl-containing compound **4** and **8** is not due to their hydrophobicity, but to their conformational rigidity and steric profile. The α-glucosidase active site possesses a narrow, elongated hydrophobic subpocket that selectively accommodates linear alkyl chains (*n*-Bu) while sterically excluding branched ones (*i*-Pr). The *n*-butyl chain in compound **5** can adopt an extended conformation to maximize van der Waals contacts, whereas the rigid, umbrella-shaped isopropyl group in compounds **4** and **8** cannot enter the pocket, resulting in no additional binding energy and no inhibitory activity.

*Conclusion on SAR.* The biological activity of compounds **2**–**9** is a direct and logical consequence of the integration of synergistic pharmacophores, depending on the form of existence (hydrochloride/base) and the fine structure of the alkyl group.

1. The hybrid structure is necessary, but not sufficient.
2. The form is critically important and acts differently for different targets (antidiabetic activity—only HCl (compounds **2** and **5**), antifungal activity—better with free base (compounds **6**–**9**).
3. The R substituent determines selectivity: Me (**2**, **6**) and *n*-Pr (**3**, **7**)→ Gram-negative microorganism (*E. coli*), *i*-Pr (**4**, **8**)→ fungi (*C. albicans*), *n*-Bu → antidiabetic.

## 3. Materials and Methods

### 3.1. Materials and Instruments

The reagents and solvents were purchased from Merck (Rahway, NJ, USA), Fluka (Taufrkirchen, Germany), Sigma-Aldrich Chemie GmbH (Darmstadt, Germany) Companies. The solvents for the synthesis, recrystallization, and TLC analysis (ethanol, *i*-PrOH, benzene, CHCl_3_, and acetone) were purified according to the standard techniques. The reported yields refer to the recrystallized samples. Product purity and reaction progress were assessed using thin-layer chromatography (TLC). Characterization of the recrystallized products involved obtaining their physicochemical data (m.p., *R*_f_), establishing the elemental composition, as well as analysis of FT-IR and NMR spectroscopic data and X-ray structural studies.

FT-IR spectra of the studied compounds were obtained on a FSM2201 spectrometer (OOO Infraspek, St. Petersburg, Russia) in KBr tablets. ^1^H- and ^13^C-NMR spectra were recorded on JNM-ECA 500 FT NMR spectrometer 500 MHz (JEOL Ltd., Akishima, Tokyo, Japan) in the deuterated solvent DMSO-d_6_. Signals of residual non-deuterated solvent DMSO were used as a standard for the ^1^H-NMR (2.50 ppm) and ^13^C-NMR (39.5 ppm) spectra. All the X-ray structural studies of single crystals were carried out on a Bruker Quest diffractometer using a Photon-III coordinate detector(Bruker AXS, Inc., Madison, WI, USA). Elemental analysis was performed on a Flash Smart elemental analyzer (ThermoFisher Scientific Inc., Waltham, MA, USA). Before recording FT-IR and NMR spectra and obtaining elemental analysis data, compounds **1**–**10** were dried for 2 h in a vacuum gun with P_2_O_5_ desiccant at 1–2 mm Hg. The melting points were determined in glass capillaries on a Stuart Melting Point Apparatus SMP30 0SA (Keison Products, Cole-Parmer group, Chelmsford, UK). The reaction progress and the purity of the products obtained were monitored using TLC Sorbfifil plates (Sorbpolymer, Krasnodar, Russia) coated with CTX-1A silica gel, grain size 5–17 μm, containing the UV-254 indicator. The eluent for TLC analysis was benzene:EtOH, 1:3. Distilled water was obtained using a medical electric water distiller AE-10 (360 V, 50 Hz, 7.2 kVA) (Livam, Belgorod, Russia).

#### 3.1.1. Synthesis of β-(Benzimidazole-1-yl)propioamidoxime (**1**)

To 11 g (0.09 mol) of benzimidazole in 30 mL of ethanol,9.55 g (0.10 mol) of acrylonitrile was added dropwise at 0–5 °C. The reaction mixture was stirred for 1 h at r.t. and then heated at reflux of ethanol for 3 h. After 24 h at r.t., 0.09 mol of a solution of hydroxylamine base in 10 mL of absolute ethanol, obtained separately from 6.25 g (0.09 mol) of hydroxylamine hydrochloride and 5.05 g (0.09 mol) of KOH after filtering off 6.71 g (0.09 mol) of KCl, was added to the reaction mixture. The reaction mixture was stirred at reflux of ethanol for 5 h under TLC monitoring. Then, after cooling to r.t., filtering from the reaction mixture and recrystallizing from i-PrOH, β-(benzimidazole-1-yl)propioamidoxime (**1**) was obtained in an amount of 10.71 g (56%): m.p. 181–183 °C; R_f_ 0.45. FT-IR (KBr, cm^−1^): 1652 (C=N), 1644 (C=C), 3462 [NO–H], 3308 [N(–H)_2_], 3157, 3152 (Csp^2^–H), 2773 and 2986 (Csp^3^–H). ^1^H-NMR (500 MHz, DMSO-d_6_, δ, ppm): 2.77 (t, J = 6.0 Hz, 2H, α-CH_2_), 4.65 (t, J = 6.0 Hz, 2H, β-CH_2_), 6.99 (s, 2H, NH_2_), 7.58–8.01 (m, 4H, Csp^2^H) and 9.61 (s, 1H, Csp^2^H). ^13^C-NMR (126 MHz, DMSO-d_6_, δ, ppm): 31.9, 42.1, 119.9, 121.9, 122.7, 134.3, 141.8, 144.5, 150.3. Anal. Calcd for C_10_H_12_N_4_O (204,23): C, 58.81; H, 5.92. Found: C, 58.53; H, 5.74. (Figures S1, S11 and S12).

#### 3.1.2. Obtaining of O-Alkylsulfonyl-β-(Benzimidazole-1-yl)propioamidoximes Hydrochlorides (**2**–**5**) Without Base Bu_3_N (***i***)

To a solution of 0.5 g (0.0024 mol) of compound **1** in 3 mL of distilled water and 15 mL of acetone, 0.0024 mol of alkylsulfonylchloride in 3 mL of acetone was added dropwise while cooling the reaction mixture to 0–5 °C and stirring; then the reaction mixture was kept at r.t.while stirring.The progress of the reaction was monitored by TLC. The solvent was evaporated under reduced pressure, and alkylsufochlorination products were obtained as amorphous powders, which were then recrystallized from *i*-PrOH and characterized using FT-IR, ^1^H-NMR, and ^13^C-NMR spectroscopy (Figures S2–S5 and S13–S20).

*O-Methylsulfonyl-β-(benzimidazole-1-yl)propioamidoxime Hydrochloride* (**2**). This compound was prepared using the general procedure described above, stirring for 20 h to provide 0.5 g (62%)of caramel-like mass **2**; *R*_f_ 0.73. FT-IR (KBr, cm^−1^): 1690 (C=N), 1655 (C=C), 1351 (SO_2_ as) and 1194 (SO_2_ sy), 3404 [N(–H)_2_], 3131 (Csp^2^–H), 2852 and 2934 (Csp^3^–H), 2700 [N(+)–H]. ^1^H-NMR (DMSO-d_6_, δ, ppm): 2.46 (s, 3H, CH_3_), 2.77 (t, J = 7.0 Hz, 2H, α-CH_2_), 4.66 (t, J = 7.0 Hz, 2H, β-CH_2_), 7.00 (s, 2H, NH_2_), 7.57–8.02 (m, 4H, Csp^2^H) and 9.61 (s, 1H, Csp^2^H). ^13^C-NMR (DMSO-d_6_, δ, ppm): 18.2, 34.8, 41.4, 113.9, 115.5, 124.5, 126.8, 131.5, 142.5, 171.6. Anal. Calcd for C_11_H_15_ClN_4_O_3_S (318.78): C, 41.45; H, 4.74. Found: C, 41.78; H, 5.13.

*O-n-Propylsulfonyl-β-(benzimidazole-1-yl)propioamidoxime Hydrochloride* (**3**). The general procedure described above was used while stirring for 16 h to provide 0.58 g (67%) of crystalline solid **3**;m.p. 89–91 °C; *R*_f_ 0.74. FT-IR (KBr, cm^−1^): 1689 (C=N), 1655 (C=C), 1357 (SO_2_ as), 1167 (SO_2_ sym), 3410 [N(-H)_2_], 3038 and 3068 (Csp^2^–H),2934 and 2968 (Csp^3^–H), 2700 [N(+)–H]. ^1^H-NMR (DMSO-d_6_, δ, ppm):0.82 (t, J = 7.5 Hz, 3H, CH_2_CH_2_CH_3_), 1.47 (m, 2H, CH_2_CH_2_CH_3_), 2.62 (t, J = 6.5 Hz, 2H, α-CH_2_), 2.90 (t, J = 7.5 Hz, 2H, CH_2_CH_2_CH_3_), 4.49 (t, J = 6.5 Hz, 2H, β-CH_2_), 6.82 (s, 2H, NH_2_), 7.25–7.65 (m, 4H, Csp^2^-H), 8.29 (s, 1H, Csp^2^-H). ^13^C-NMR (DMSO-d_6_, δ, ppm): 16.3, 31.3, 41.1, 49.0, 110.9, 119.8, 122.0, 134.1, 143.8, 144.6, 157.5. Anal. Calcd for C_13_H_19_ClN_4_O_3_S (346.83): C, 45.02; H, 5.52. Found: C, 45.50; H, 5.13.

*O-Isopropylsulfonyl-β-(benzimidazole-1-yl)propioamidoxime Hydrochloride* (**4**). The general procedure described above was used while stirring for 20 h to provide 0.5 g (58%) of crystalline solid **4**; m.p. 163–165 °C; *R*_f_ 0.75. FT-IR (KBr, cm^−1^): 3415 [N(-H)_2_], 3043 (Csp^2^–H), 2938, 2875 (Csp^3^–H), 2700 [N(+)–H], 1690 (C=N), 1654 (C=C), 1347 (SO_2_ as), 1179 (SO_2_ sym). ^1^H-NMR (DMSO-d_6_, δ, ppm): δ 1.04 (d, J = 7.0 Hz, 6H, CH(CH_3_)_2_), 2.60 (t, J = 7.0 Hz, 2H, α-CH_2_), 3.11 (m, 1H, CH(CH_3_)_2_), 4.46 (t, J = 7.0 Hz, 2H, β-CH_2_), 6.92 (m, 2H, NH_2_), 7.17–7.60 (m, 4H, Csp^2^H), 8.12 (s, 1H, Csp^2^H). ^13^C-NMR (DMSO-d_6_, δ, ppm): 16.2, 31.4, 40.2, 48.9, 110.92, 119.4, 122.0, 122.8, 143.8, 144.5, 144.0, 157.4. Anal. Calcd for C_13_H_19_ClN_4_O_3_S (346.83): C, 45.02; H, 5.52. Found: C, 45.42; H, 5.91.

*O-n-Butylsulfonyl-β-(benzimidazole-1-yl)propioamidoxime Hydrochloride*(**5**). After stirring for 20 h, 0.36 g (40%) of compound **5** was obtained as a crystalline powder; m.p. 82–84 °C; *R*_f_ 0.74. FT-IR (KBr, cm^−1^): 3460 [N(-H)_2_], 3060 (Csp^2^–H), 2962, 2871 (Csp^3^–H), 2750 [N(+)–H], 1691 (C=N), 1655 (C=C), 1355 (SO_2_ as), 1165 (SO_2_ sym). ^1^H-NMR (DMSO-d_6_, δ, ppm): 0.77 (t, J = 7.0 Hz, 3H, CH_2_CH_2_CH_2_CH_3_), 1.22 (m, 2H, CH_2_CH_2_CH_2_CH_3_), 1.43 (m, 2H, CH_2_CH_2_CH_2_CH_3_), 2.78 (t, J = 6.5 Hz, 2H, α-CH_2_), 2.98 (t, J = 7.0 Hz, 2H, CH_2_CH_2_CH_2_CH_3_), 4.75 (t, J = 6.5 Hz, 2H, β-CH_2_), 7.00 (m, 2H, NH_2_), 7.57–8.07 (m, 4H, Csp^2^H), 9.61 (s, 1H, Csp^2^H), ^13^C-NMR (DMSO-d_6_, δ, ppm): 13.9, 21.1, 25.3, 30.5, 44.0, 47.6, 113.8, 115.5, 126.4, 126.7, 131.6, 142.4, 157.4. Anal. Calcd for C_14_H_21_ClN_4_O_3_S (360.86): C, 46.60; H, 5.87. Found: C, 46.78; H, 5.69.

#### 3.1.3. Obtaining of O-Alkylsulfonyl-β-(Benzimidazole-1-yl)propioamidoximes Bases (**6**–**9**) (***ii***)

To a solution of 0.5 g O-alkylsulfonyl-β-(benzimidazole-1-yl)propioamidoxime hydrochlorides (**2**–**5**) in 5 mL of distilled water, an equivalent amount of potash was added at r.t.while stirring. The reaction mixture was stirred for 1 h under TLC monitoring, and then the amorphous technical precipitates of the bases **6**–**9** were filtered and recrystallized from *i*-PrOH and characterized using FT-IR, ^1^H-NMR, and ^13^C-NMR spectroscopy (Figures S6–S9 and S21–S28).

*O-Methylsulfonyl-β-(benzimidazole-1-yl)propioamidoxime* (**6**). The above-described method allowed to obtain 0.37 g (83%) of compound **6** as a white solid; m.p. 144–146 °C; *R*_f_ 0.79. FT-IR (KBr, cm^−1^): 3431 [N(-H)_2_], 3128 (Csp^2^–H), 2976, 2943 (Csp^3^–H), 1664 (C=N), 1499 (C=C), 1347 (SO_2_ as), 1191 (SO_2_ sym). ^1^H-NMR (DMSO-d_6_, δ, ppm): 2.29 (s, 3H, SO_2_CH_3_), 2.90 (t, J = 7.0 Hz, 2H, α-CH_2_), 4.49 (t, J = 7.0 Hz, 2H, β-CH_2_), 6.82 (s, 2H, NH_2_), 7.19–7.66 (m, 4H, Csp^2^H), 8.29 (s, 1H, Csp^2^H). ^13^C-NMR (DMSO-d_6_, δ, ppm): δ 17.1, 31.21, 41.9, 111.2, 119.4, 122.3, 123.3, 133.0, 142.3, 144.3, 157.5. Anal. Calcd for C_11_H_14_N_4_O_3_S (282.32): C, 46.80; H, 5.00. Found: C, 47.15; H, 5.37.

*O-n-Propylsulfonyl-β-(benzimidazole-1-yl)propioamidoxime* (**7**). Using the above-described method, 0.35 g (77%) of compound 7 as a white solid was obtained; m.p. 139–141 °C; *R*_f_ 0.78. FT-IR (KBr, cm^−1^): 3431 [N(-H)_2_], 3126, 3032 (Csp^2^–H), 2976 (Csp^3^–H), 1664 (C=N), 1498 (C=C), 1347 (SO_2_ as), 1192 (SO_2_ sym). ^1^H-NMR (DMSO-d_6_, δ, ppm): 0.81 (t, J = 7.0 Hz, 3H, CH_2_CH_2_CH_3_), 1.45 (m, 2H, CH_2_CH_2_CH_3_), 2.59 (t, J = 7.0 Hz, 2H, CH_2_CH_2_CH_3_), 2.90 (t, J = 6.0 Hz, 2H, α-CH_2_), 4.47 (t, J = 6.0 Hz, 2H, β-CH_2_), 6.81 (s, 2H, NH_2_), 7.14–7.61 (m, 4H, Csp^2^H), 8.14 (s, 1H, Csp^2^H). ^13^C-NMR (DMSO-d_6_, δ, ppm): 12.5, 16.6, 31.5, 35.9, 41.7, 110.9, 119.9, 122.0, 122.8, 134.2, 143.8, 144.6, 157.9. Anal. Calcd for C_13_H_18_N_4_O_3_S (310.37): C, 50.31; H, 5.85. Found: C, 50.67; H, 5.46.

*O-Isopropylsulfonyl-β-(benzimidazole-1-yl)propioamidoxime* (**8**). Using the above-described method, 0.30 g (66%) of compound **8** was obtained as white crystals; m.p. 158–160 °C; *R*_f_ 0.79. FT-IR (KBr, cm^−1^): 3452 [N(–H)_2_], 3153, 3110 (Csp^2^–H), 2993, 2973 (Csp^3^–H), 1655 (C=N), 1503 (C=C), 1341 (SO_2_ as), 1178 (SO_2_ sym). ^1^H-NMR (DMSO-d_6_, δ, ppm): δ 1.02 (d, J = 7.0 Hz, 6H, CH(CH_3_)_2_), 2.61 (t, J = 6.5 Hz, 2H, α-CH_2_), 3.11 (m, 1H, CH(CH_3_)_2_), 4.47 (t, J = 6.5 Hz, 2H, β-CH_2_), 6.82 (s, 2H, NH_2_), 7.16–7.81 (m, 4H, Csp^2^H), 8.12 (s, 1H, Csp^2^H). ^13^C-NMR (DMSO-d_6_, δ, ppm): 16.4, 31.3, 41.7, 49.0, 110.0, 119.1, 121.9, 122.8, 134.2, 143.5, 144.5, 157.5. Anal. Calcd for C_13_H_18_N_4_O_3_S (310.37): C, 50.31; H, 5.85. Found: C, 50.77; H, 5.45.

*O-n-Butylsulfonyl-β-(benzimidazole-1-yl)propioamidoxime* (**9**). The above-described method allowed to obtain 0.30 g (68%) of compound **9** as a white solid; m.p.130–132 °C; *R*_f_ 0.75. FT-IR (KBr, cm^−1^): 3408 [N(-H)_2_], 3057 (Csp^2^–H), 2958, 2874 (Csp^3^–H), 1662 (C=N), 1499 (C=C), 1347 (SO_2_ as), 1159 (SO_2_ sym). ^1^H-NMR (DMSO-d_6_, δ, ppm): 0.79 (t, J = 7.5 Hz, 3H, CH_2_CH_2_CH_2_CH_3_), 1.41 (m, 2H, CH_2_CH_2_CH_2_CH_3_), 1.45 (m, 2H, CH_2_CH_2_CH_2_CH_3_), 2.60 (t, J = 6.5 Hz, 2H, α-CH_2_), 2.94 (t, J = 7.5 Hz, 2H, CH_2_CH_2_CH_2_CH_3_), 4.46 (t, J = 6.5 Hz, 2H, β-CH_2_), 6.82 (s, 2H, NH_2_), 7.16–7.57 (m, 4H, Csp^2^H), 8.12 (s, 1H, Csp^2^H). ^13^C-NMR (DMSO-d_6_, δ, ppm): δ 13.9, 21.1, 25.4, 31.4, 41.7, 47.5, 110.9, 119.9, 121.9, 122.8, 134.2, 143.8, 144.6, 157.6. Anal. Calcd for C_14_H_20_N_4_O_3_S (324.40): C, 51.83; H, 6.21. Found: C, 51.65; H, 6.63.

#### 3.1.4. Interaction of Alkyl Sulfochlorides with β-(Benzimidazol-1-yl)propioamidoxime (**1**) Using the Base Bu_3_N (***iii***)

The alkyl substituents in the series of alkylsulfonyl chlorides AlkSO_2_Cl were: methyl, *n*-propyl, *i*-propyl, *n*-butyl. The reaction was carried out in CHCl_3_ at r.t.; Bu_3_N was used as a base to bind the HCl released in the reaction. Under these reaction conditions, amidoxime hydrochloride (**10**) and amidoxime (**1**) were obtained as the products in all the cases in a ratio of approximately 60% and 20%. The obtained products **1** and **10** were characterized using FT-IR, ^1^H-NMR, and ^13^C-NMR spectroscopy (Figures S1, S10–S12, S29 and S30).

*Interaction of Isopropylsulfonyl Chloride with β-(Benzimidazol-1-yl)propioamidoxime (**1**) in the presence of Bu_3_N.* To a solution of 0.5 g (0.0024 mol) of β-(benzimidazole-1-yl)propioamidoxime (**1**) in 20 mL of CHCl_3_, a solution of 0.44 g (0.0024 mol) of Bu_3_N in 3 mL of CHCl_3_ was added. Cooling of the reaction mixture with an ice bath to 0–5 °C was used during the dropwise addition of a solution of 0.34 g (0.0024 mol) of isopropylsulfonyl chloride in 3 mL of CHCl_3_; then the reaction mixture was kept at r.t. The reaction progress was monitored by TLC. The reaction time was 20 h. After completion of the reaction, 0.35 g (61%) of β-(benzimidazol-1-yl)propioamidoxime hydrochloride (**10**) with a m.p. of 171–173 °C and *R*_f_ 0.66 was obtained after recrystallization from *i*-PrOH. FT-IR (KBr, cm^−1^): 1690 (C=N), 1637 (C=C), 3277 [N(–H)_2_], 2370 [N(+)–H]. ^1^H-NMR (DMSO-d_6_, δ, ppm): 3.12 (t, J = 6.5 Hz, 2H, α-CH_2_), 4.98 (t, J = 6.5 Hz, 2H, β-CH_2_), 7.56–8.12 (m, 4H, Csp^2^H), 8.65 and 9.54 (two s, 2H, NH_2_), 9.77 (s, 1H, Csp^2^H), 11.48 (10.82 [br.s, exchangeable protons of the groups N(+)H and NOH]. ^13^C-NMR (DMSO-d_6_, δ, ppm): 29.3, 50.0, 113.8, 115.6, 126.6, 126.8, 131.5, 142.3, 144.0, 159.3. Anal. Calcd for C_10_H_13_ClN_4_O (240.69): C, 49.90; H, 5.44. Found: C, 50.28; H, 5.23.

The starting amidoxime **1** was isolated from the filtrate by evaporation of the reaction mixture after recrystallisation from *i*-PrOH with a yield of 0.1 g (21%), m.p. 181–183 °C and *R*_f_ 0.45. FT-IR (KBr, cm^−1^): 1695 (C=N), 1655 (C=C), 3400 [N(–H)_2_], 3420 (NO–H). ^1^H-NMR (DMSO-d_6_, δ, ppm): 2.77 (t, J = 6.0 Hz, 2H, α-CH_2_), 4.65 (t, J = 7.0 Hz, 2H, β-CH_2_), 6.99 (s, 2H, NH_2_), 7.55–8.01 (m, 4H, Csp^2^H) and 9.61 (s, 1H, Csp_2_H). ^13^C-NMR (DMSO-d_6_, δ, ppm): 31.9, 42.1, 119.9, 121.9, 122.7, 134.3, 141.8, 144.5, 150.3. Anal. Calcd for C_10_H_12_N_4_O (204.23): C, 58.81; H, 5.92. Found: C, 58.38; H, 5.63.

### 3.2. Single-Crystal X-Ray Diffraction

All X-ray diffraction studies of single crystals were carried out on a Bruker ApexII diffractometer using a two-coordinate CCD detector. The structures were solved using the dual-space method and the Patterson minimum superposition function (SHELXT program [44]). The structures were refined in the full-matrix least-squares anisotropic approximation for all the non-hydrogen atoms. The *n*-propyl and *n*-butyl groups in the structures of **3** and **5** are disordered over two positions with equal probability. The positions of the H(N) and H(O) atoms were located from difference Fourier maps, and those of the H(C) atoms were placed in calculated positions. All the hydrogen atoms were refined isotropically using a riding model with restraints on bond lengths. All the calculations for structure refinement were performed using the SHELXL [45] and OLEX2 GUI version [46] programs.

### 3.3. The In Vitro Biological Screening

#### 3.3.1. In Vitro Antimicrobial and Antifungal Screening

Antimicrobial and antifungal activities were evaluated using a broth microdilution method to determine the minimum inhibitory concentration (MIC, µg/mL). Testing was performed against *Staphylococcus aureus* ATCC 6538, *Bacillus subtilis* ATCC 6633, *Escherichia coli* ATCC 25922, *Pseudomonas aeruginosa* ATCC 27853, and *Candida albicans* ATCC 10231, with gentamicin and nystatin as reference standards.

Compounds were tested in a range of 1.56 to 100 µg/mL. Microbial suspensions (~10^6^ CFU/mL) were incubated with the compounds at 37 °C for 24–48 h. The MIC was defined as the lowest concentration with no visible growth. Controls confirmed the solvent (≤1% ethanol) did not affect growth. The results are the average of three independent experiments [47].

#### 3.3.2. In Vitro α-Glucosidase Inhibition Assay

α-Glucosidase inhibitory activity was assessed using a modified literature method [47]. Test compounds and the acarbose standard were dissolved in DMSO. The assay, performed in phosphate buffer (pH 6.8), involved pre-incubating the enzyme with the compound at 37 °C, followed by initiation with the substrate *p*-nitrophenyl-α-D-glucopyranoside (pNPG). The reaction was stopped with sodium carbonate after 20 min. Due to high initial absorbance, the samples were diluted five-fold with a solution of 0.1 M sodium carbonate before measuring the absorbance at 405 nm. Inhibition was calculated relative to a DMSO control. All the tests were performed in triplicate.

## 4. Conclusions

This study successfully synthesized novel O-alkylsulfonyl-β-(benzimidazol-1-yl)propioamidoximes (**2**–**9**). A key finding was that base-free conditions in water-acetone yielded the target hydrochlorides (**2**–**5**), utilizing the substrate’s own benzimidazole nitrogen to bind HCl. Conversely, using an external base (Bu_3_N) failed, resulting only in recovered starting material and β-(benzimidazol-1-yl)propioamidoxime hydrochloride due to competitive reactions of alkylsulfonyl chlorides with external base.

The X-ray analysis definitively identified the benzimidazole Nsp^2^ atom as the protonation site in the O-alkylsulfonyl-β-(benzimidazol-1-yl)propioamidoximes hydrochlorides.

The work yielded two key outcomes: (1) potent antifungal agents (the isopropylsulfonyl derivatives **4** and **8**) and (2) dual-action hybrids (methyl-**2** and *n*-butylsulfonyl **5** derivatives) with superior antidiabetic activity and broad antimicrobial/antifungal profiles.

In conclusion, integrating benzimidazole, amidoxime, and sulfonyl pharmacophores created highly active compounds—O-alkylsulfonyl-β-(benzimidazol-1-yl)propioamidoximes, validating this scaffold for developing new multi-target therapeutics.

## Figures and Tables

**Table 1:** Physicochemical data of the starting amidoxime **1**, O-alkylsulfochlorination products **2**–**9** and β-(benzimidazol-1-yl)propioamidoxime hydrochloride **10**.

Comp	Alk	Yield, %	Time, h	M.p., °C	*R*_f_	Comp	Alk	Yield, %	Time, h	M.p., °C	*R*_f_
**1**	-	56 (21 *)	20	181–183	0.45	**6**	Me	83	1	144–146	0.79
**2**	Me	62	20	-	0.73	**7**	*n*-Pr	77	1	139–141	0.78
**3**	*n*-Pr	67	16	89–91	0.74	**8**	*i*-Pr	66	1	158–160	0.79
**4**	*i*-Pr	58	20	163–165	0.75	**9**	*n*-Bu	68	1	130–132	0.75
**5**	*n*-Bu	40	20	82–84	0.74	**10**	-	61	20	171–173	0.66

* The yield of amidoxime **1** in the process of alkylsulfochlorination under conditions (***iii***).

**Table 2:** In vitro antimicrobial and antifungal activity (MIC, µg/mL) and α-glucosidase antidiabetic inhibition (%) of the O-alkylsulfonyl-β-(benzimidazole-1-yl)propioamidoximes (**2**–**9**).

Compd	Alk	Gram-Positive Strains		Gram-Negative Strains		*Candida* *albicans*	α-Glucosidase Inhibition, % *
		*Staphylococcus aureus*	*Bacillus* *subtilis*	*Escherichia coli*	*Pseudomonas aeruginosa*		
2	Me	-	50	12.5	-	25	64.7 ± 3.1
3	*n*-Pr	-	-	25	-	25	-
4	*i*-Pr	25	-	-	-	12.5	-
5	*n*-Bu	12.5	25	-	-	50	79.5 ± 0.2
6	Me	-	50	12.5	-	25	-
7	*n*-Pr	50	-	25	-	-	-
8	*i*-Pr	12.5	-	-	-	6.3	-
9	*n*-Bu	25	-	-	-	-	-
Gentamicin		6.3	6.3	3.1	6.3	-	-
Nistatin		-	-	-	-	12.5	-
Acarbose		-	-	-	-	-	44.7 ± 1.0

* Significance of differences *p* < 0.05 compared with the comparison group.

## Data Availability

The original contributions presented in this study are included in the article/Supplementary Materials. Further inquiries can be directed to the corresponding author.

## Supplementary Materials

The following supporting information can be downloaded at https://www.mdpi.com/article/10.3390/ijms27146224/s1.
