# Supplementary material for: Structural Modifications of Hybrid O-Alkylsulfonyl-β-(Benzimidazol-1-yl)propioamidoximes as a Pathway to the Antimicrobial, Antifungal and Antidiabetic Drugs
Source: Int J Mol Sci. 2026 Jul 12;27(14):6224. doi: 10.3390/ijms27146224 (PMC13411167; doi:10.3390/ijms27146224)
Supplement: Supplementary file 1 [file ijms-27-06224-s001.zip › ijms-4356856-supplementary.pdf]

Supplementary Data

Spectral Data of β-(Benzimiazol-1-yl)propioamidoxime and its O-Alkylsufochlorination Products

I. FT-IR Spectra of the Compounds 1–10 (KBr, cm^{-1})

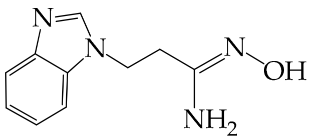

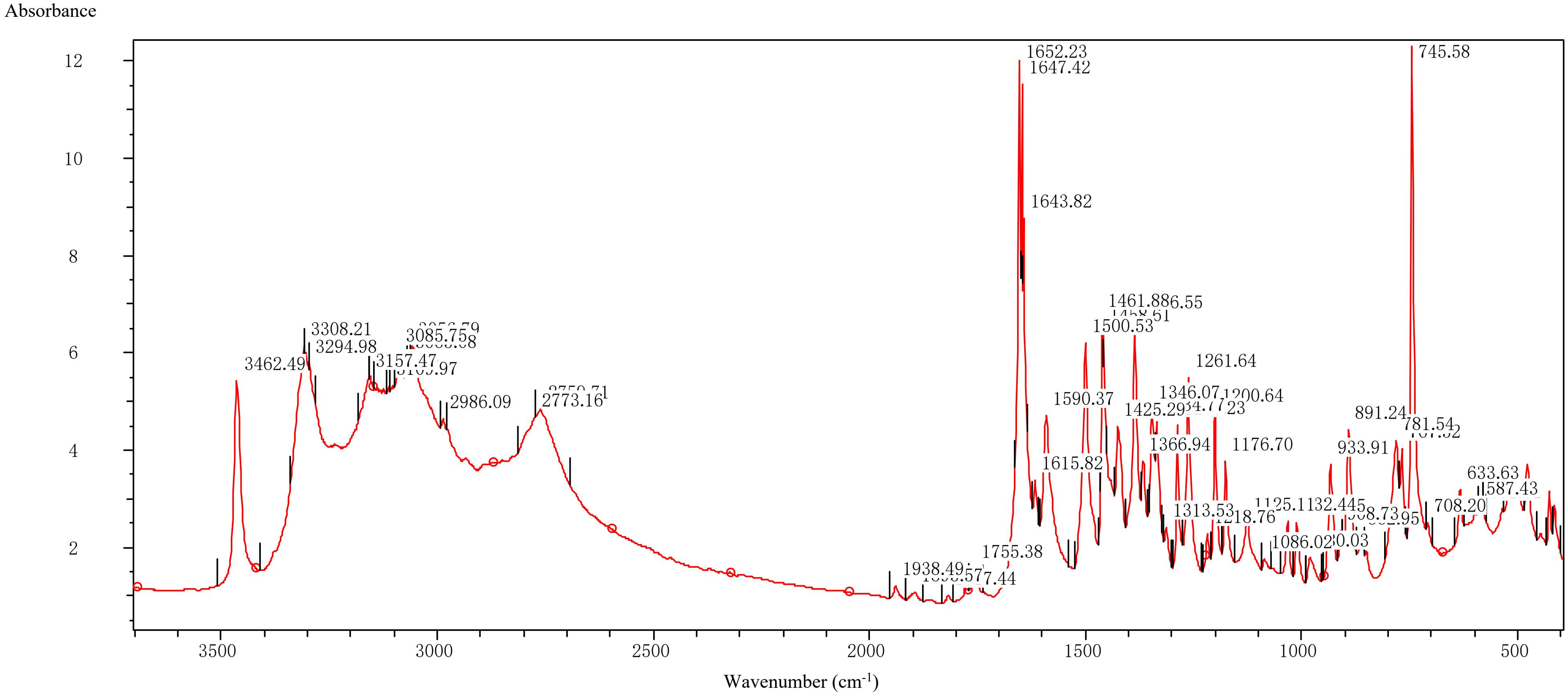

Figure S1. FT-IR spectrum of β-(benzimiazol-1-yl)propioamidoxime (**1**).

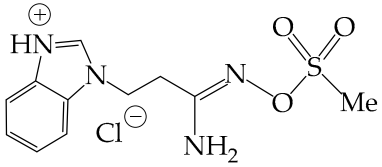

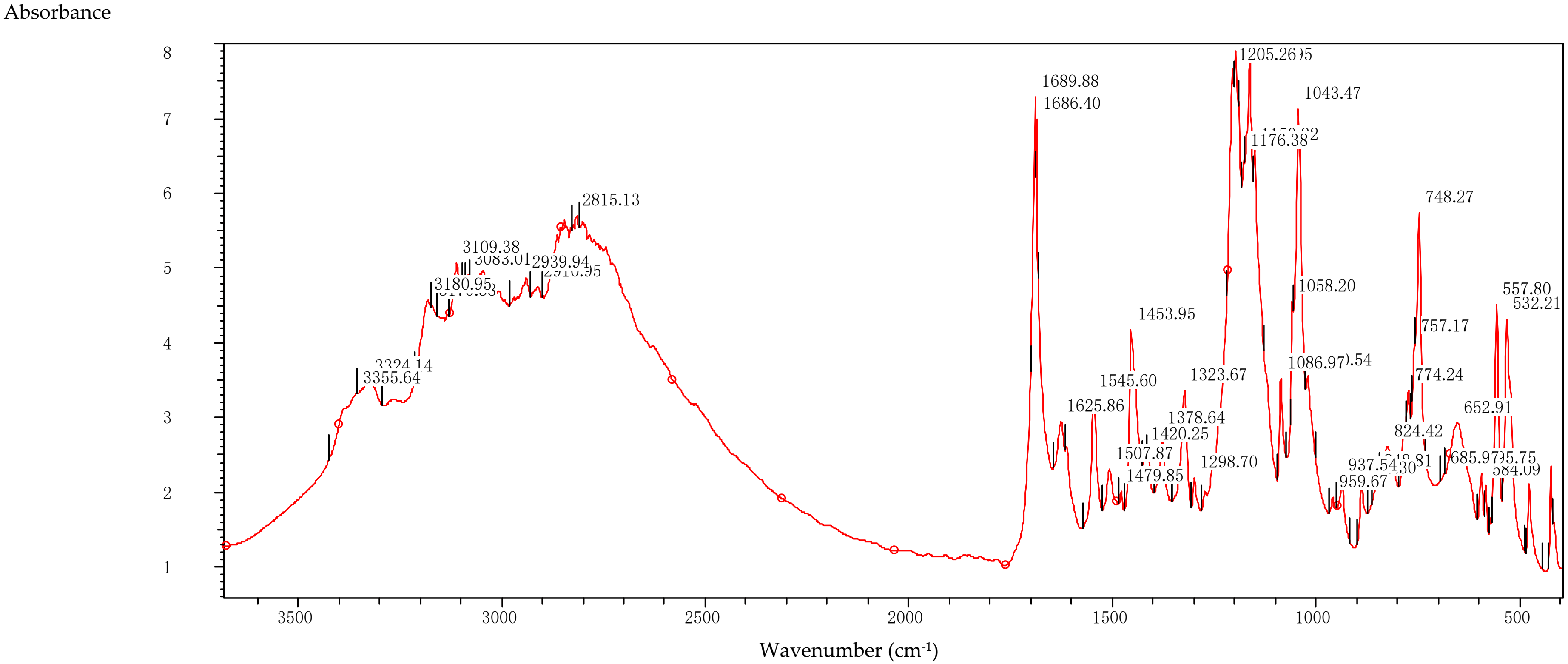

Figure S2. FT-IR spectrum of O-methylsulfonyl-β-(benzimidazole-1-yl)propioamidoxime hydrochloride (**2**).

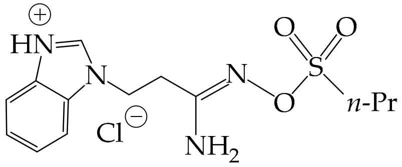

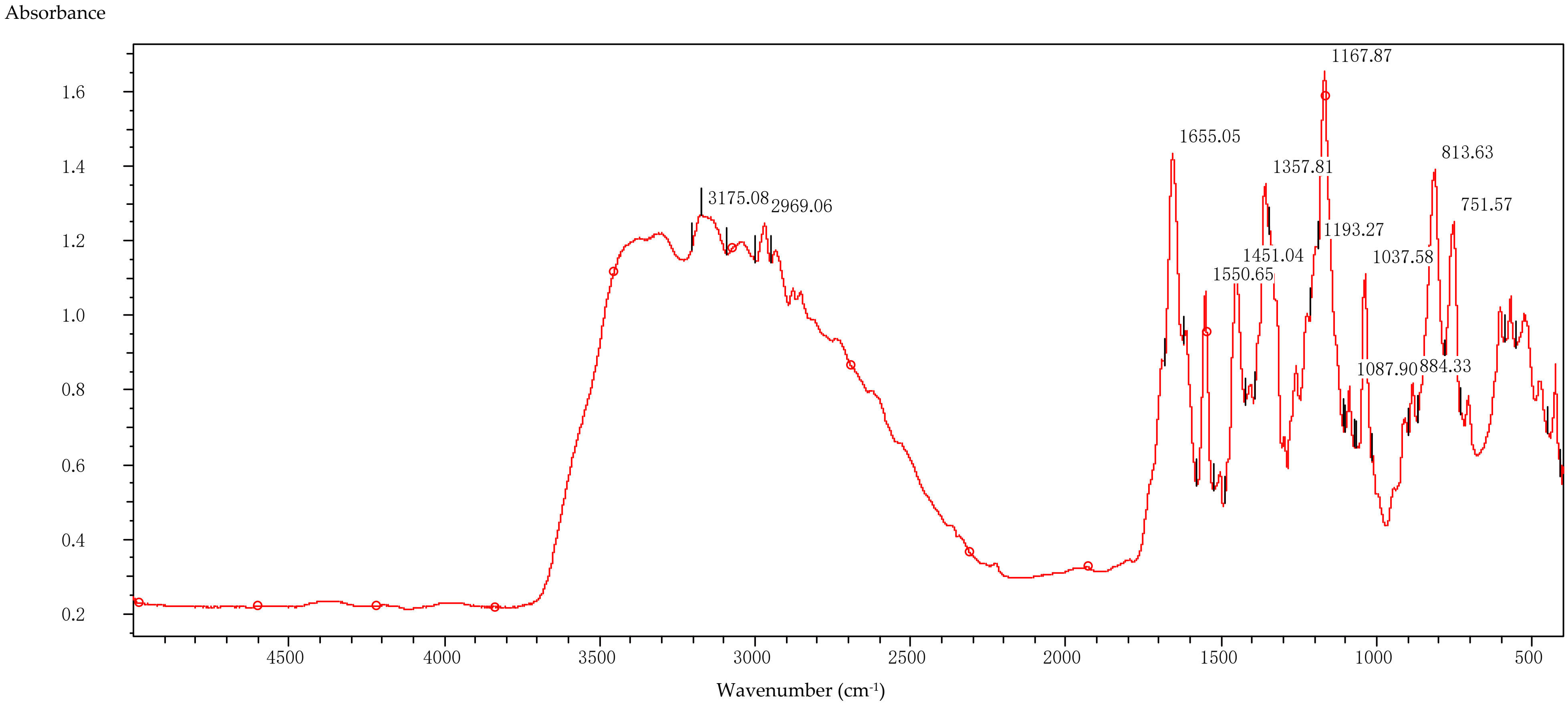

Figure S3. FT-IR spectrum of O-*n*-propylsulfonyl-β-(benzimidazole-1-yl)propioamidoxime hydrochloride (**3**).

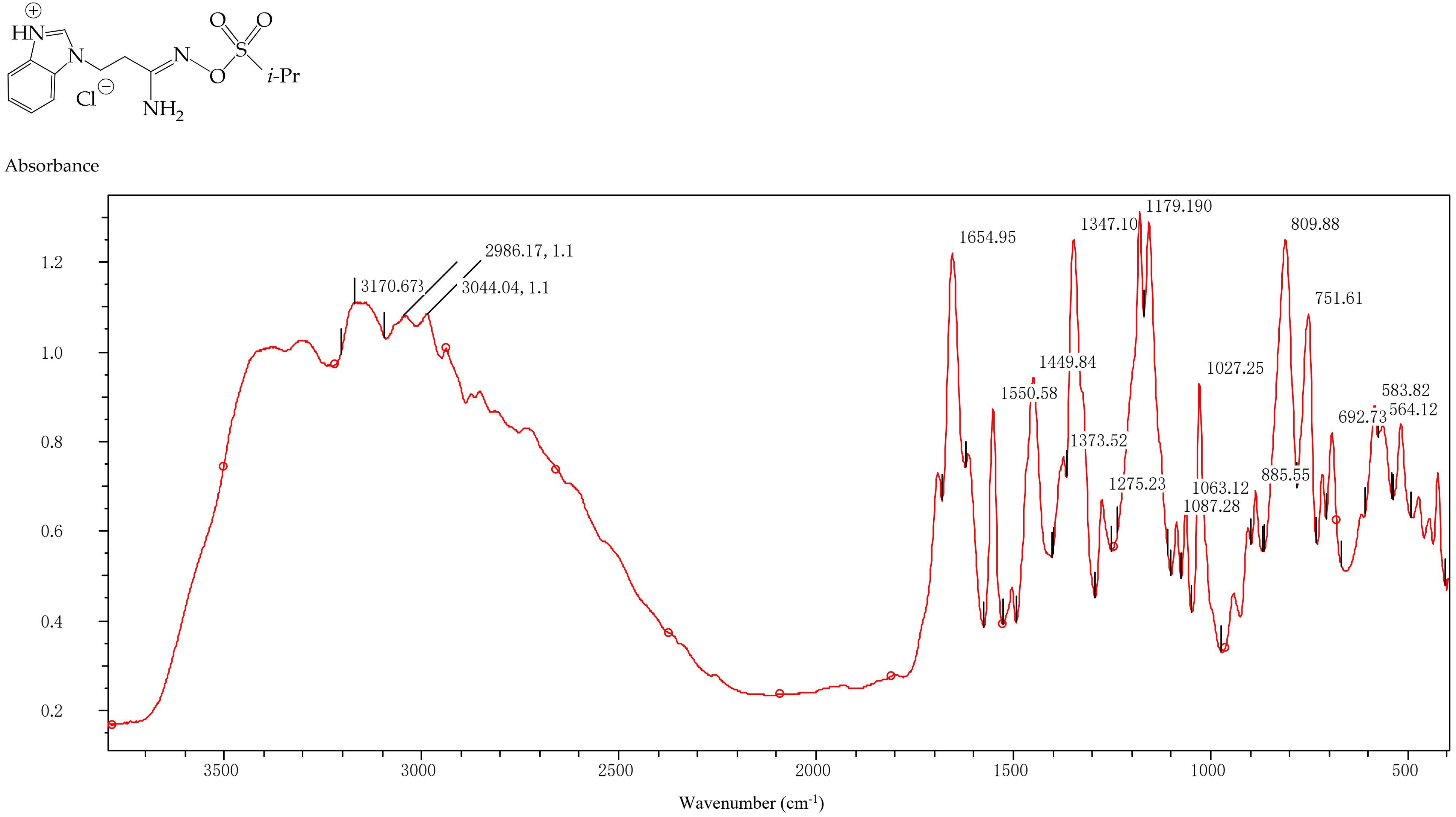

Figure S4. FT-IR spectrum of O-isopropylsulfonyl-β-(benzimidazole-1-yl)propioamidoxime hydrochloride (**4**).

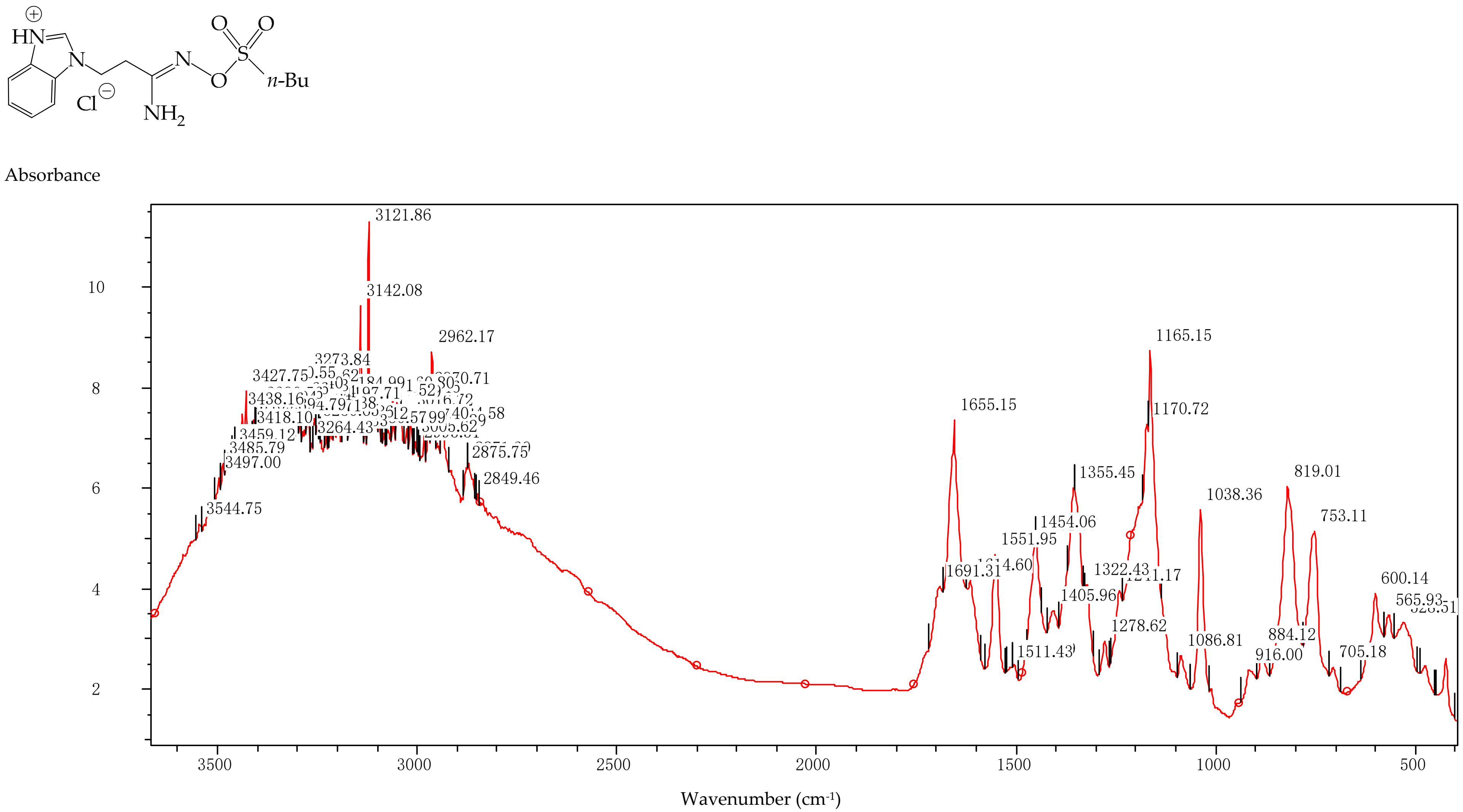

Figure S5. FT-IR spectrum of O-*n*-butylsulfonyl-β-(benzimidazole-1-yl)propioamidoxime hydrochloride (**5**).

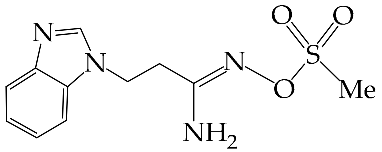

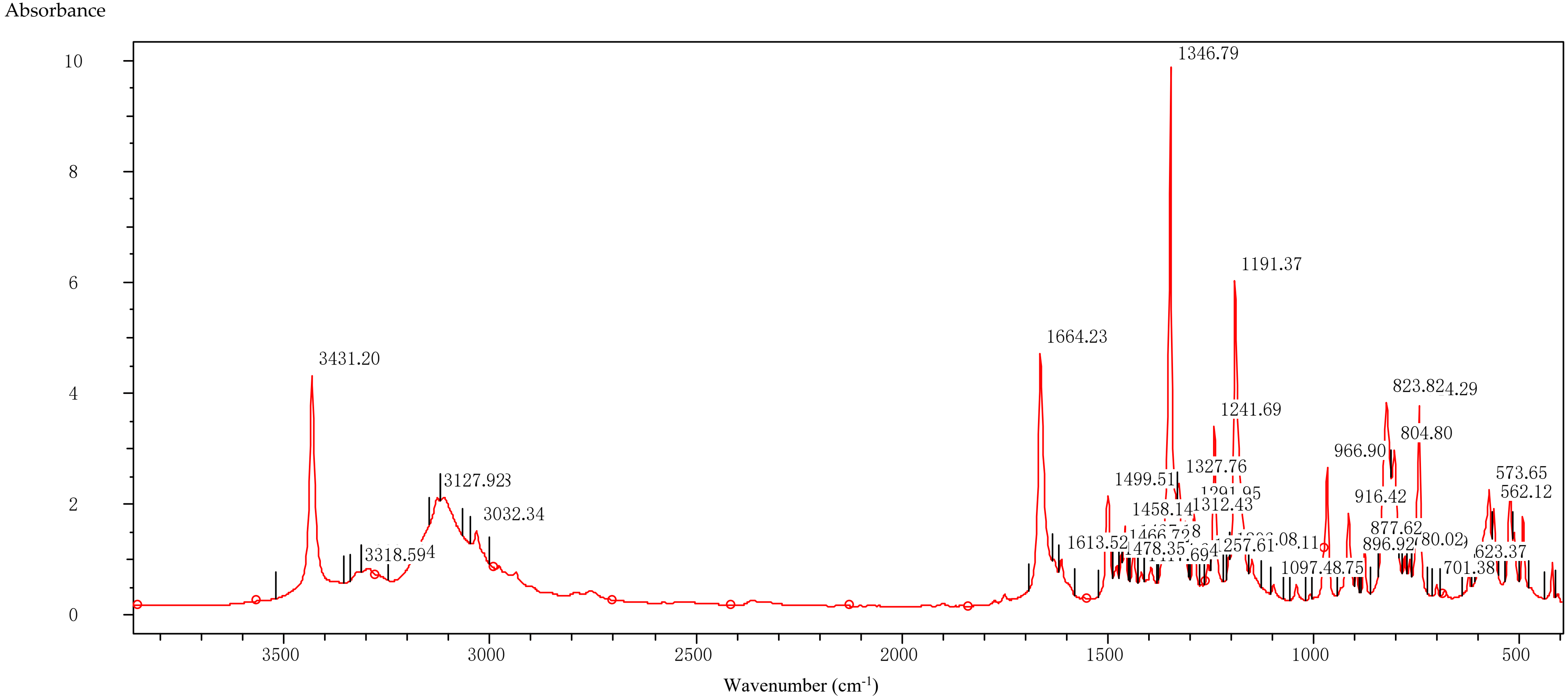

Figure S6. FT-IR spectrum of O-methylsulfonyl-β-(benzimidazole-1-yl)propioamidoxime (**6**).

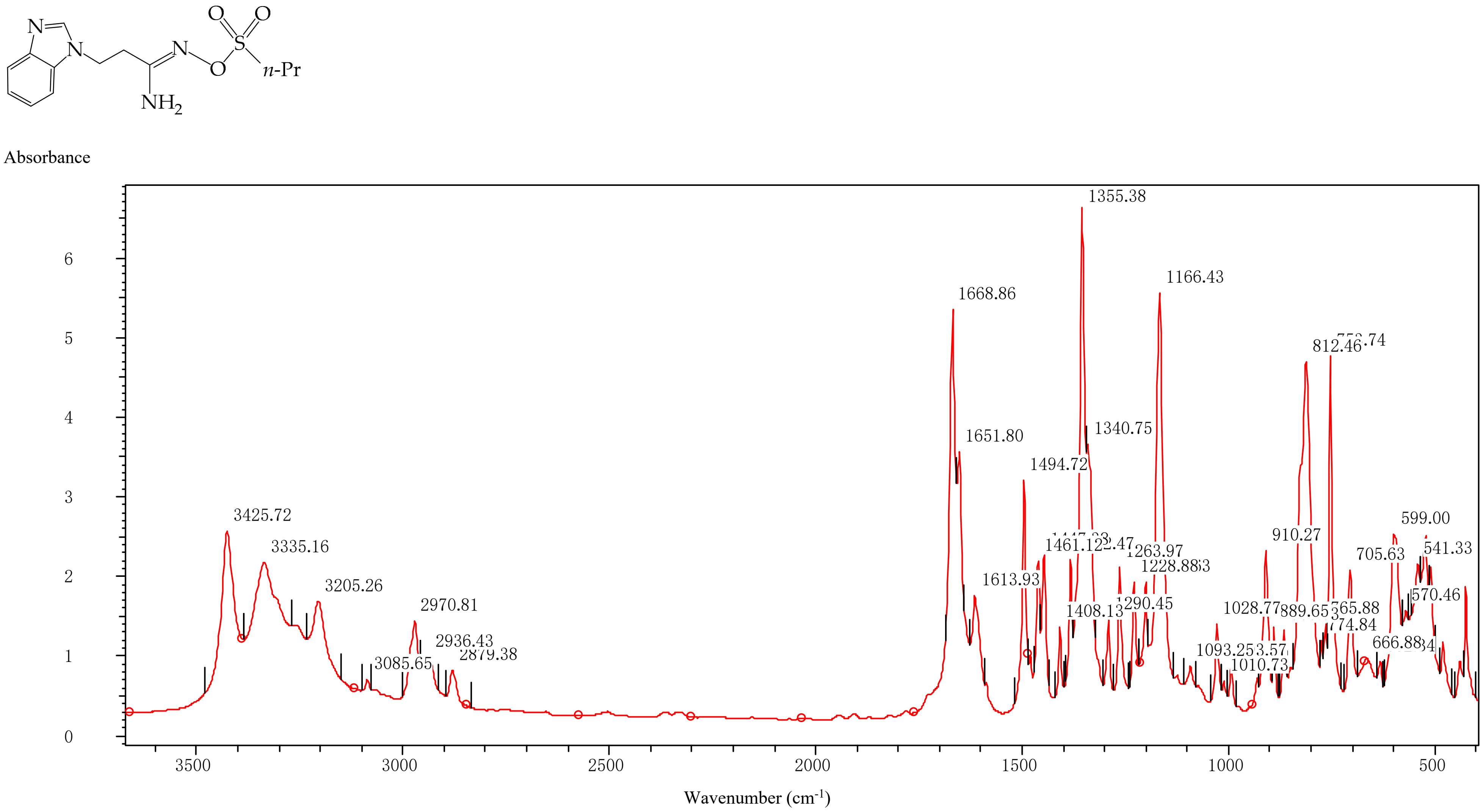

Figure S7. FT-IR spectrum of O-*n*-propylsulfonyl-β-(benzimidazole-1-yl)propioamidoxime (**7**).

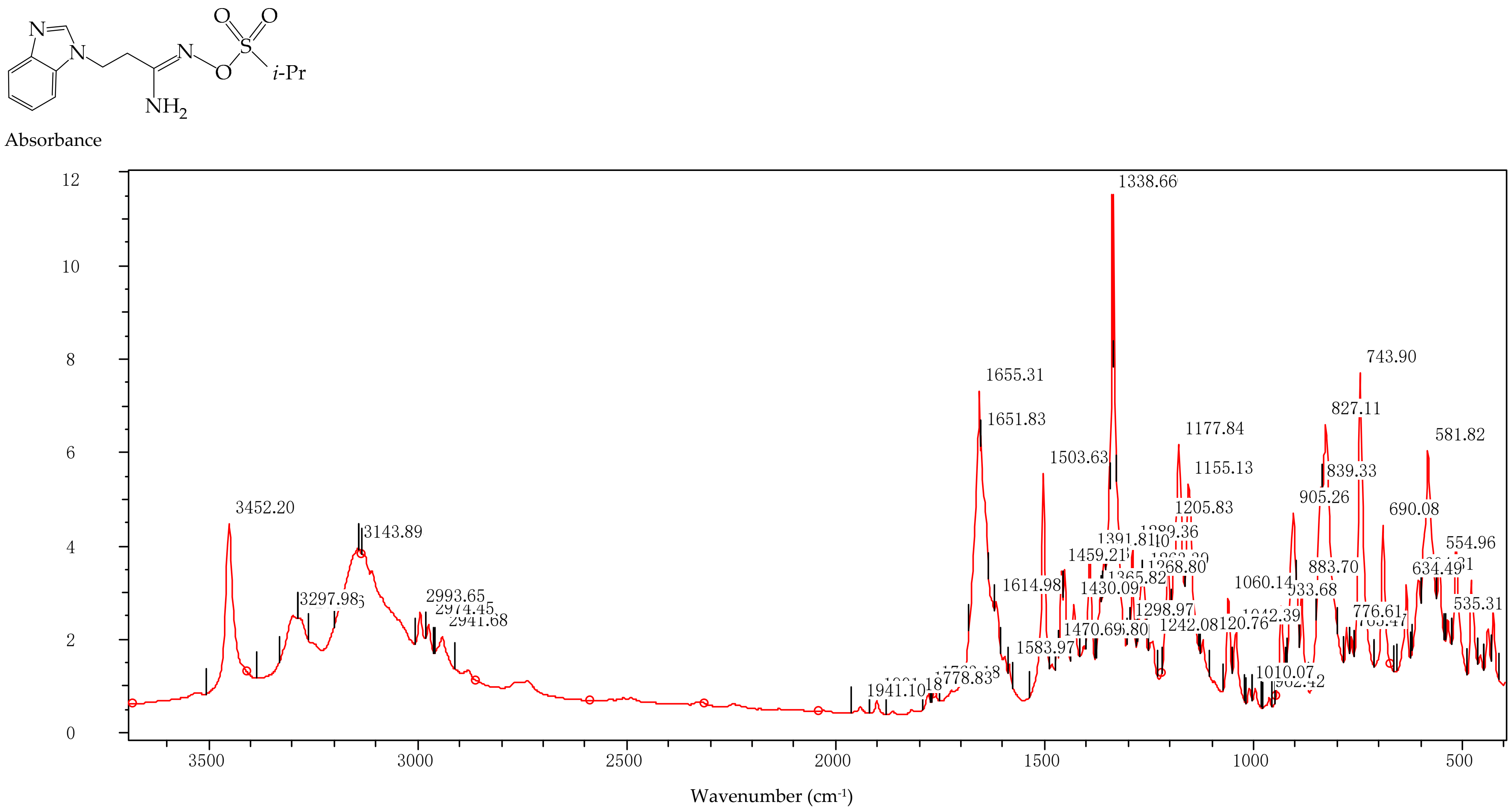

Figure S8. FT-IR spectrum of O-isopropylsulfonyl-β-(benzimidazole-1-yl)propioamidoxime (**8**).

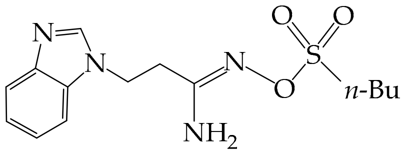

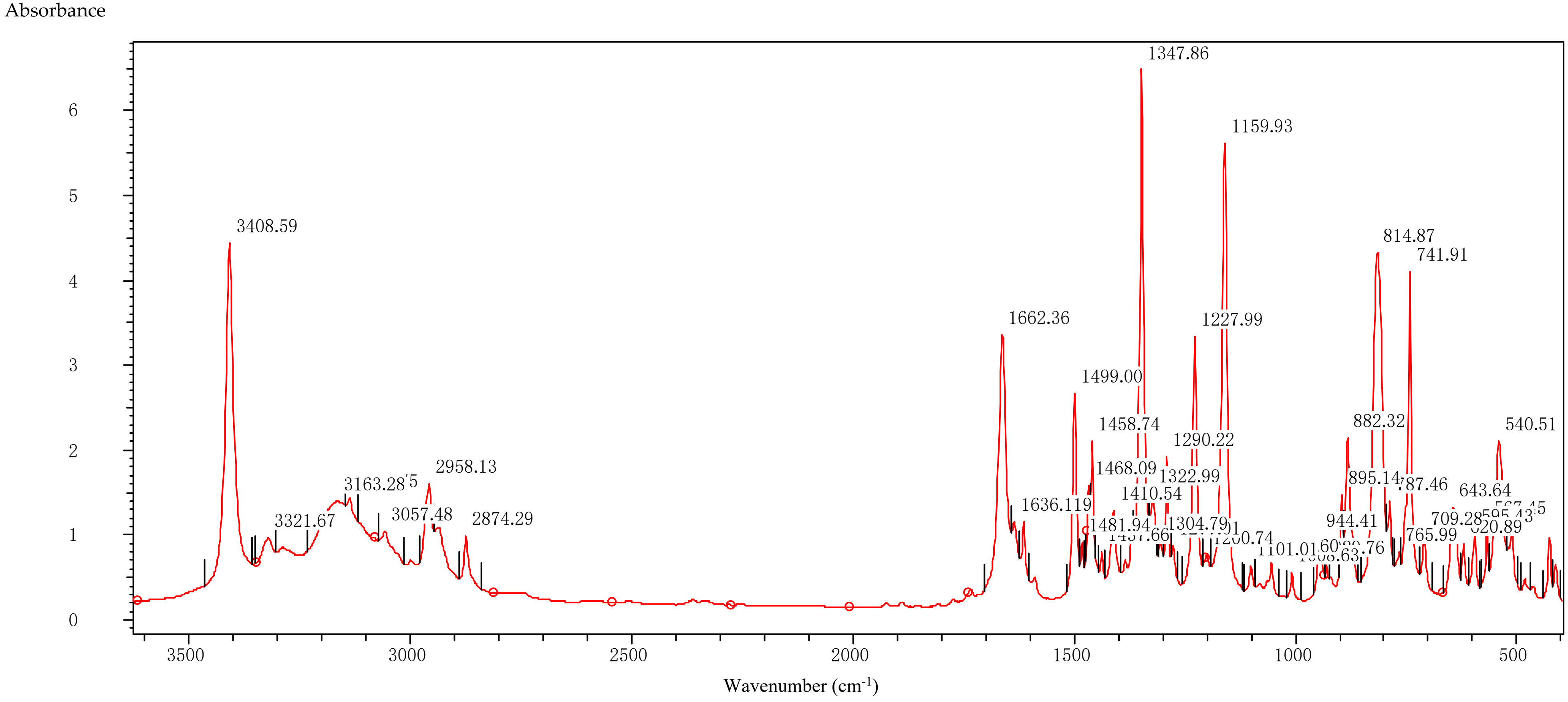

Figure S9. FT-IR spectrum of O-*n*-butylsulfonyl-β-(benzimidazole-1-yl)propioamidoxime (**9**).

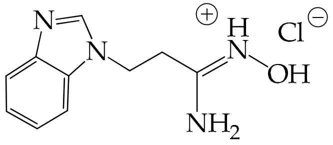

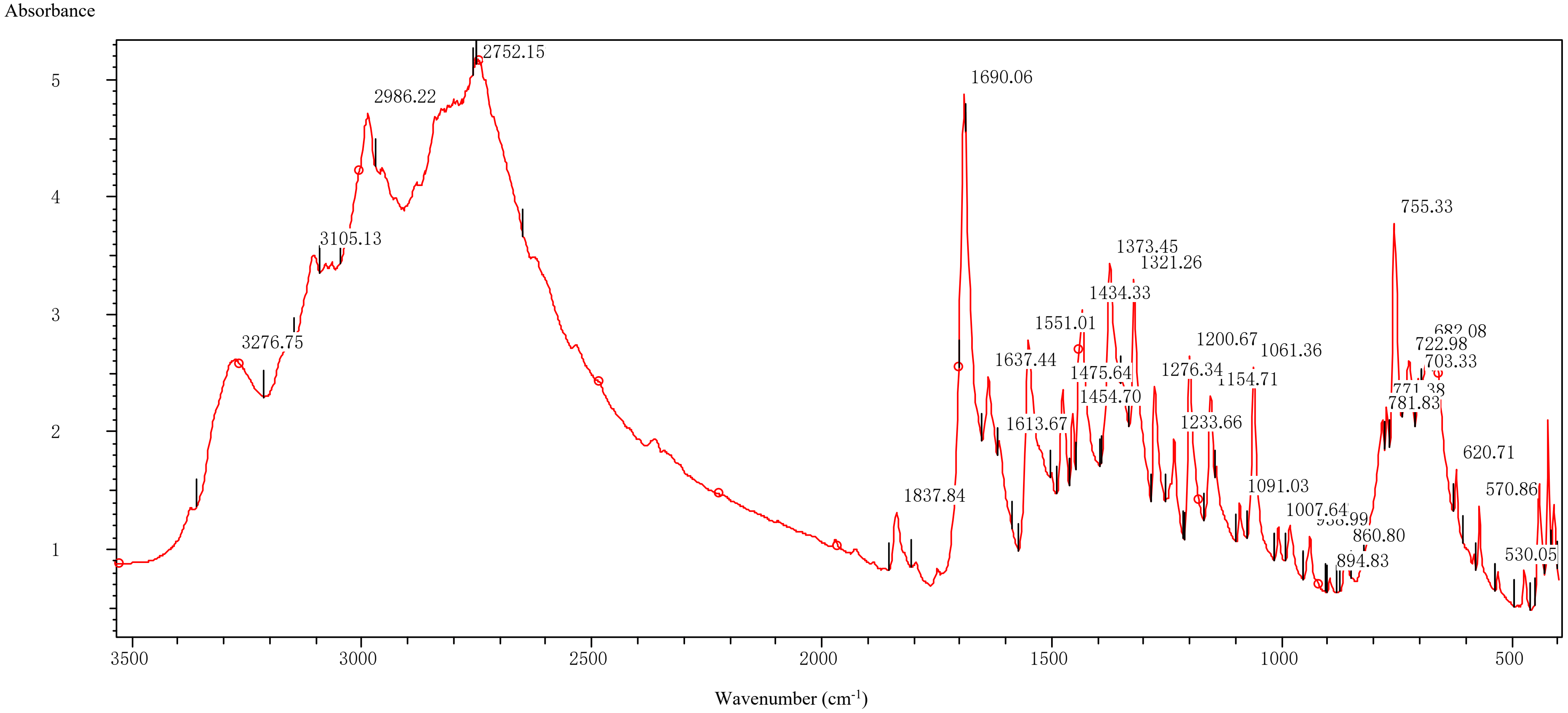

Figure S10. FT-IR spectrum of β-(benzimidazole-1-yl)propioamidoxime hydrochloride (**10**).

II. ^{1}H and ^{13}C-NMR Spectra of the Compounds 1–10, δ, ppm (DMSO-*d*$_6$)

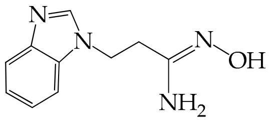

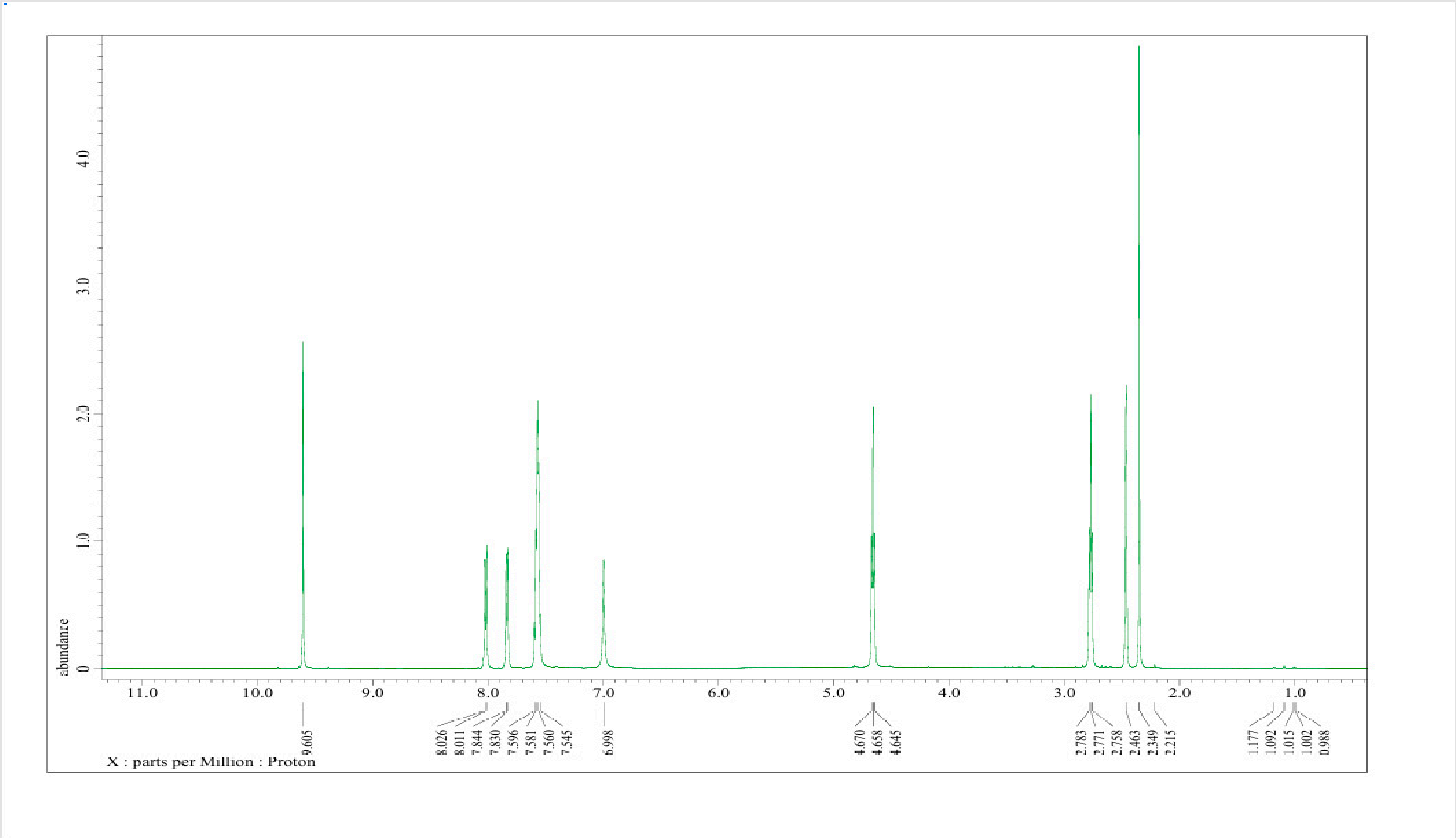

Figure S11. ^{1}H NMR spectrum of β-(benzimidazole-1-yl)propioamidoxime (**1**).

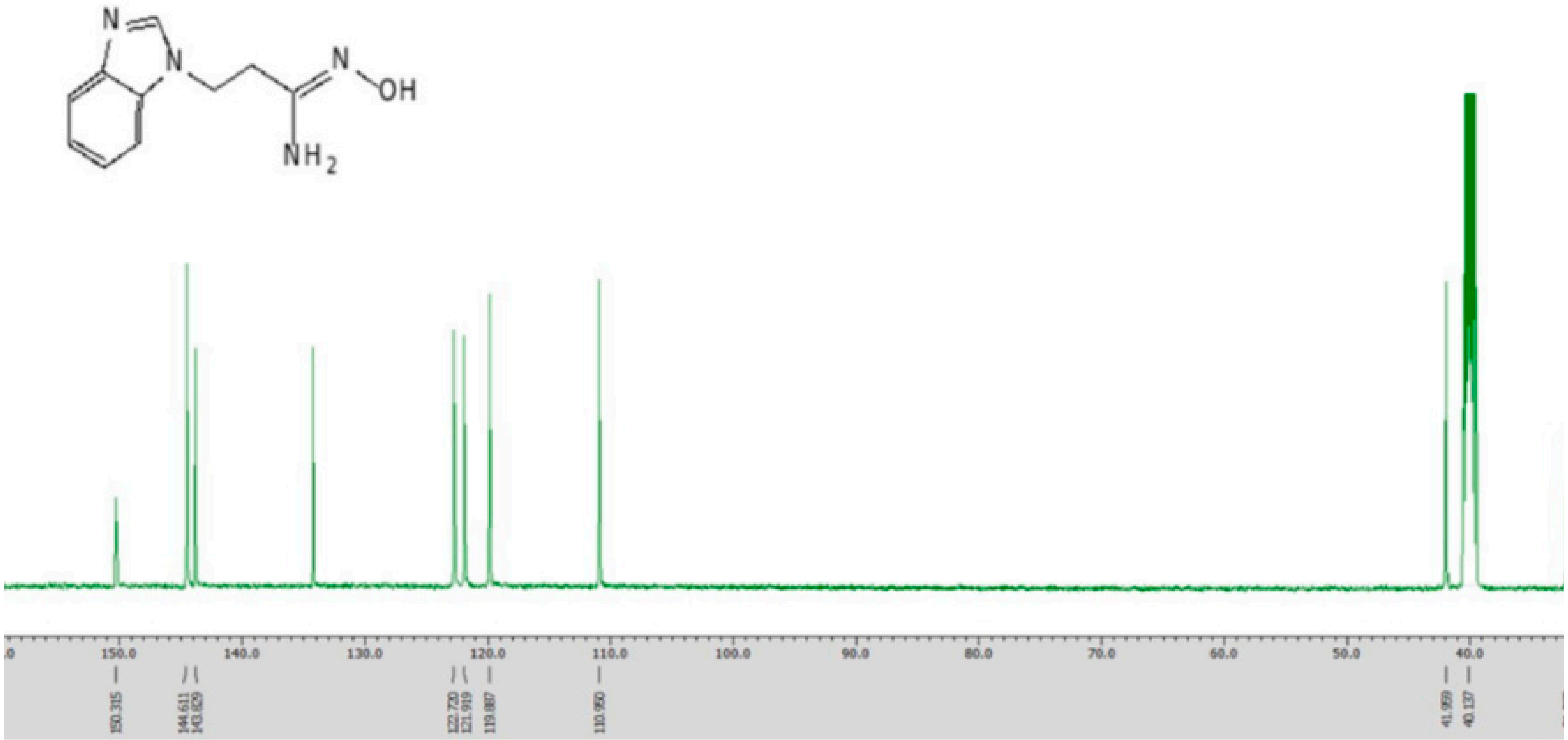

Figure S12. ^{13}C NMR spectrum of β-(benzimidazole-1-yl)propioamidoxime (**1**).

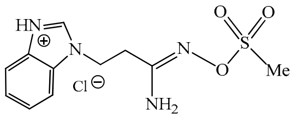

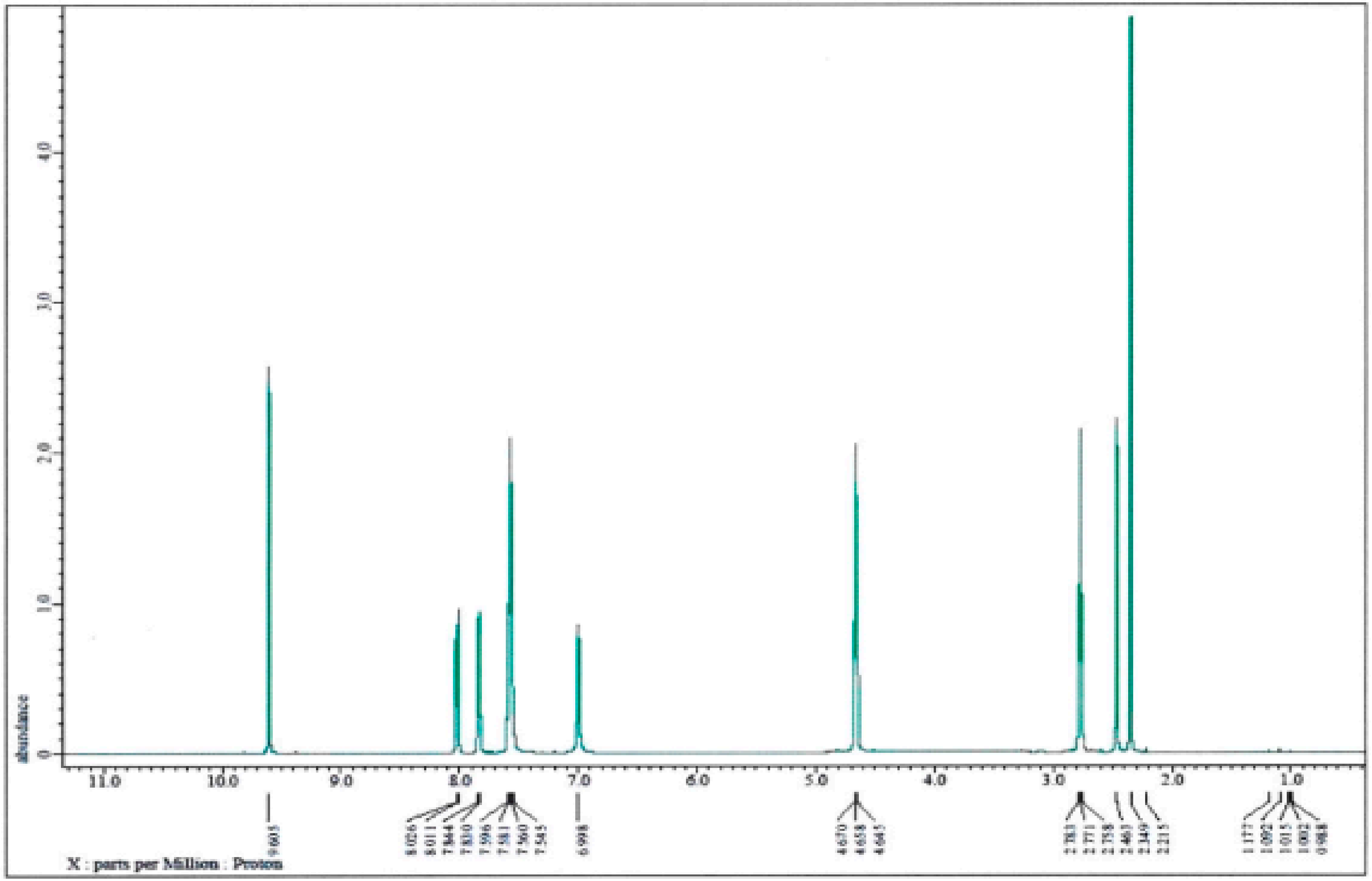

Figure S13. ^{1}H NMR spectrum of O-methylsulfonyl-β-(benzimidazole-1-yl)propioamidoxime hydrochloride (**2**).

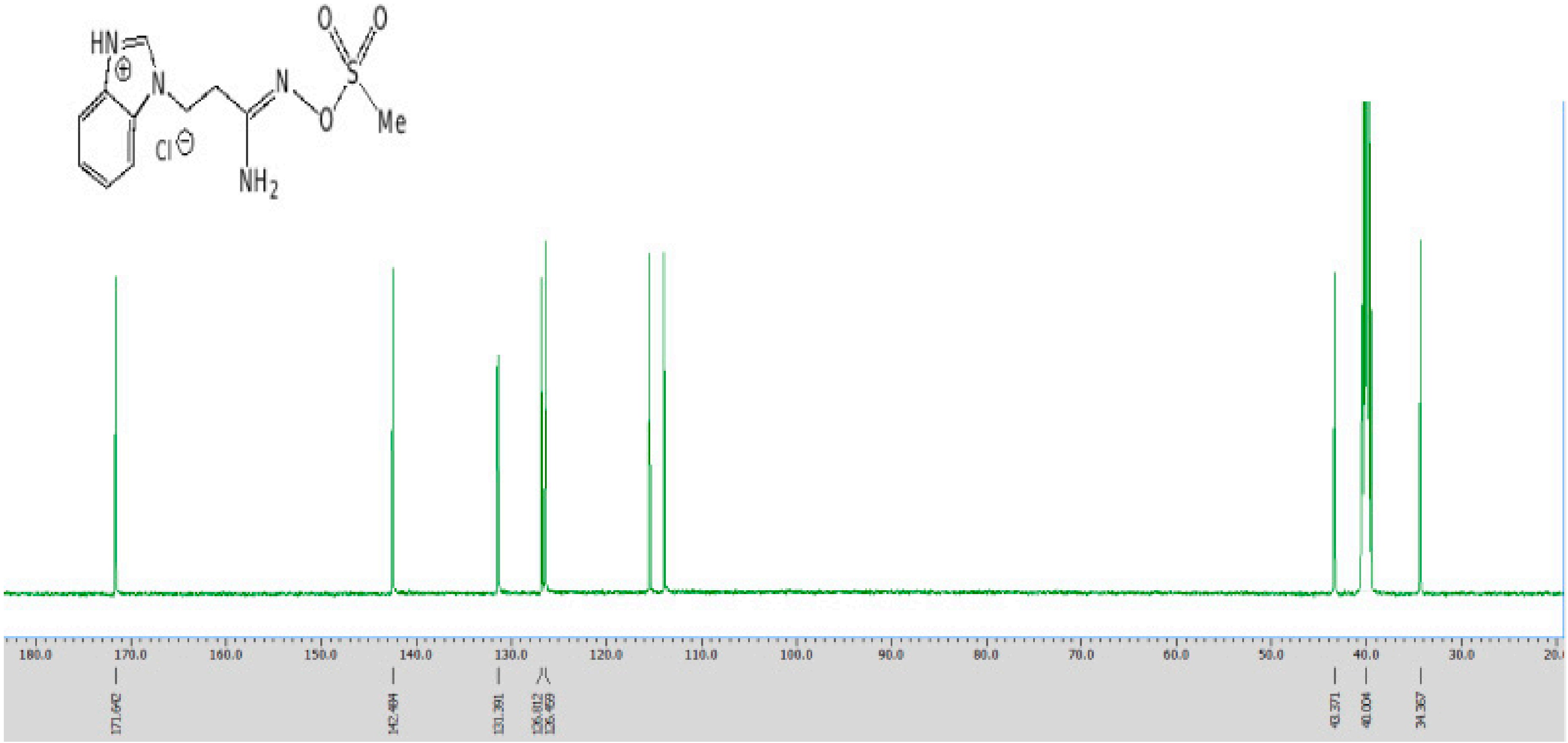

Figure S14. ^{13}C NMR spectrum of O-methylsulfonyl-β-(benzimidazole-1-yl)propioamidoxime hydrochloride (**2**).

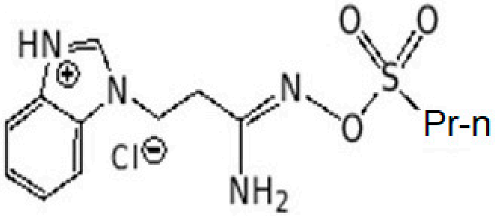

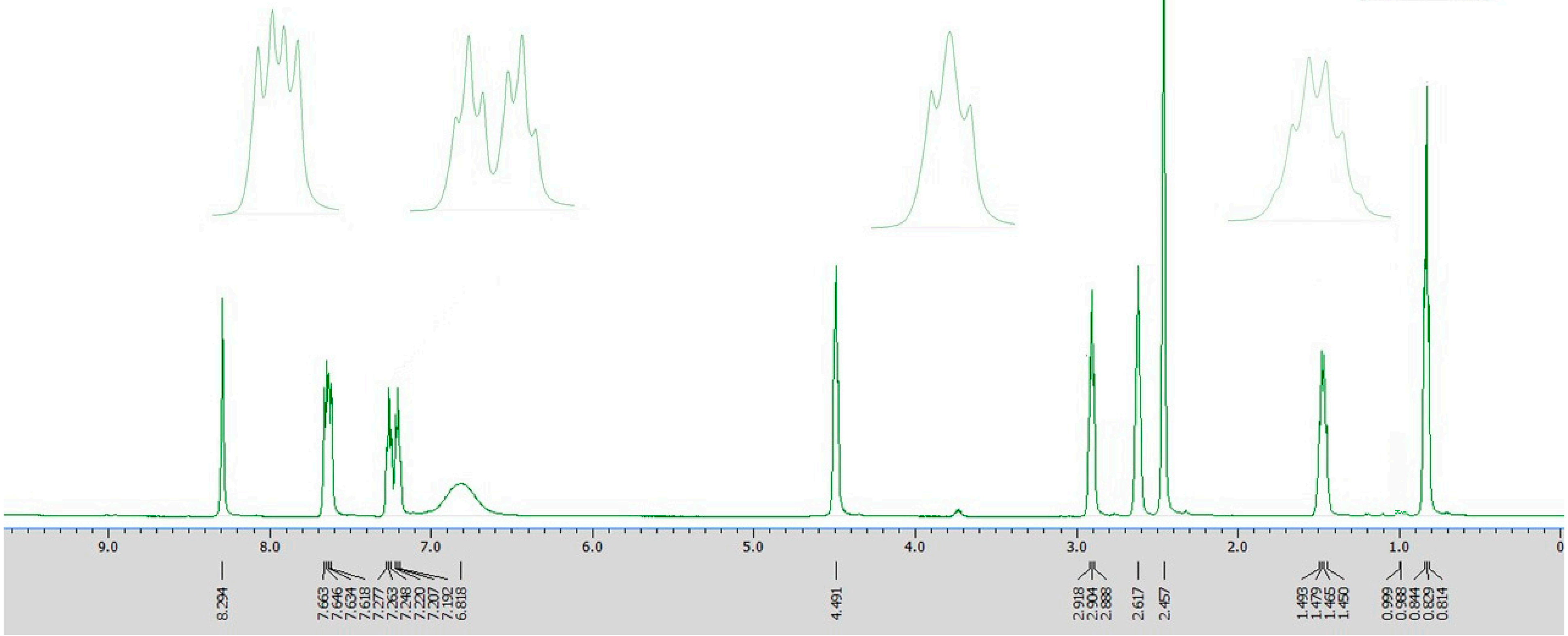

Figure S15. ^{1}H NMR spectrum of O-*n*-propylsulfonyl-β-(benzimidazole-1-yl)propioamidoxime hydrochloride (**3**).

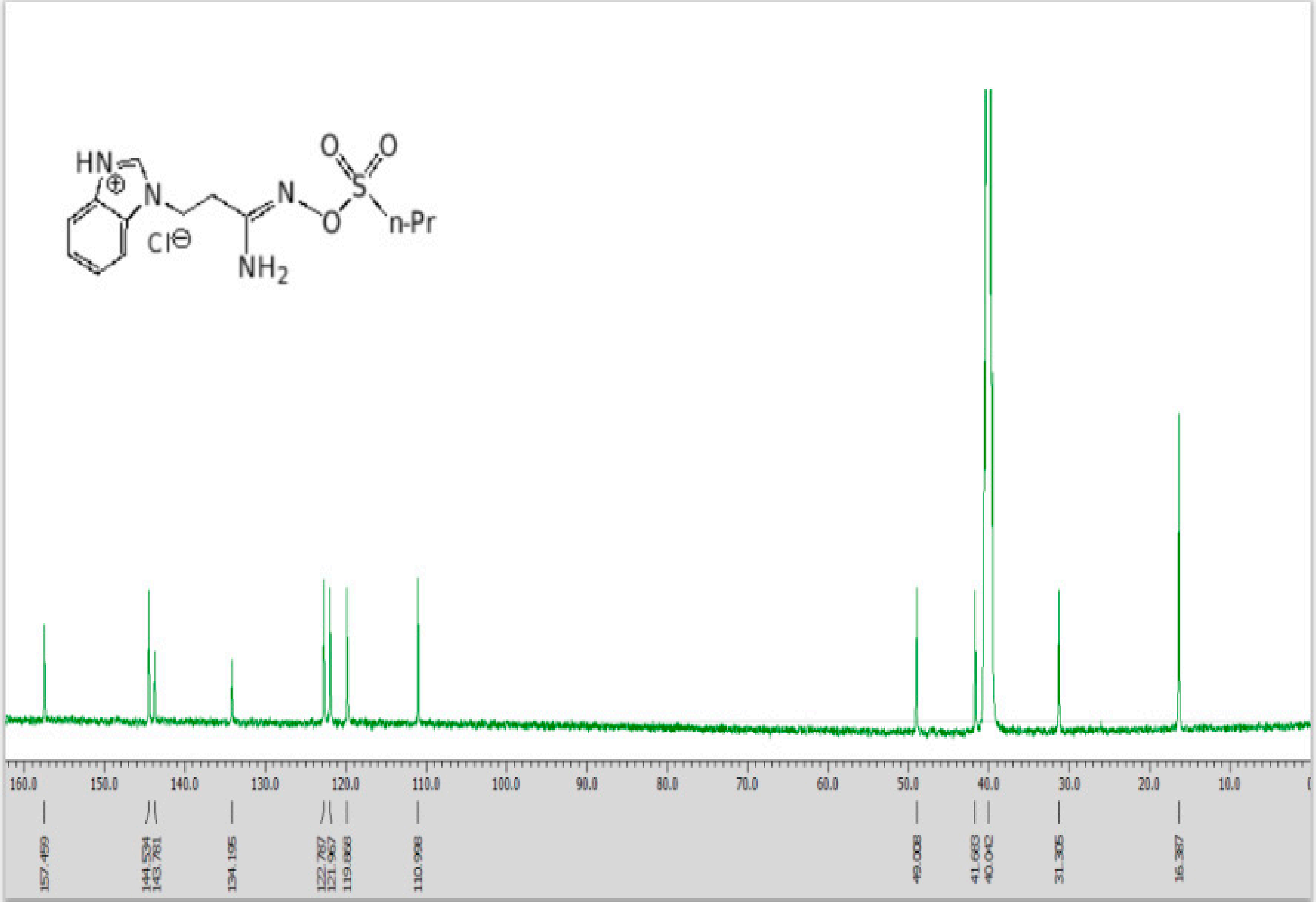

Figure S16. ^{13}C NMR spectrum of O-*n*-propylsulfonyl-β-(benzimidazole-1-yl)propioamidoxime hydrochloride (**3**).

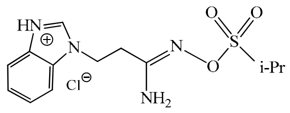

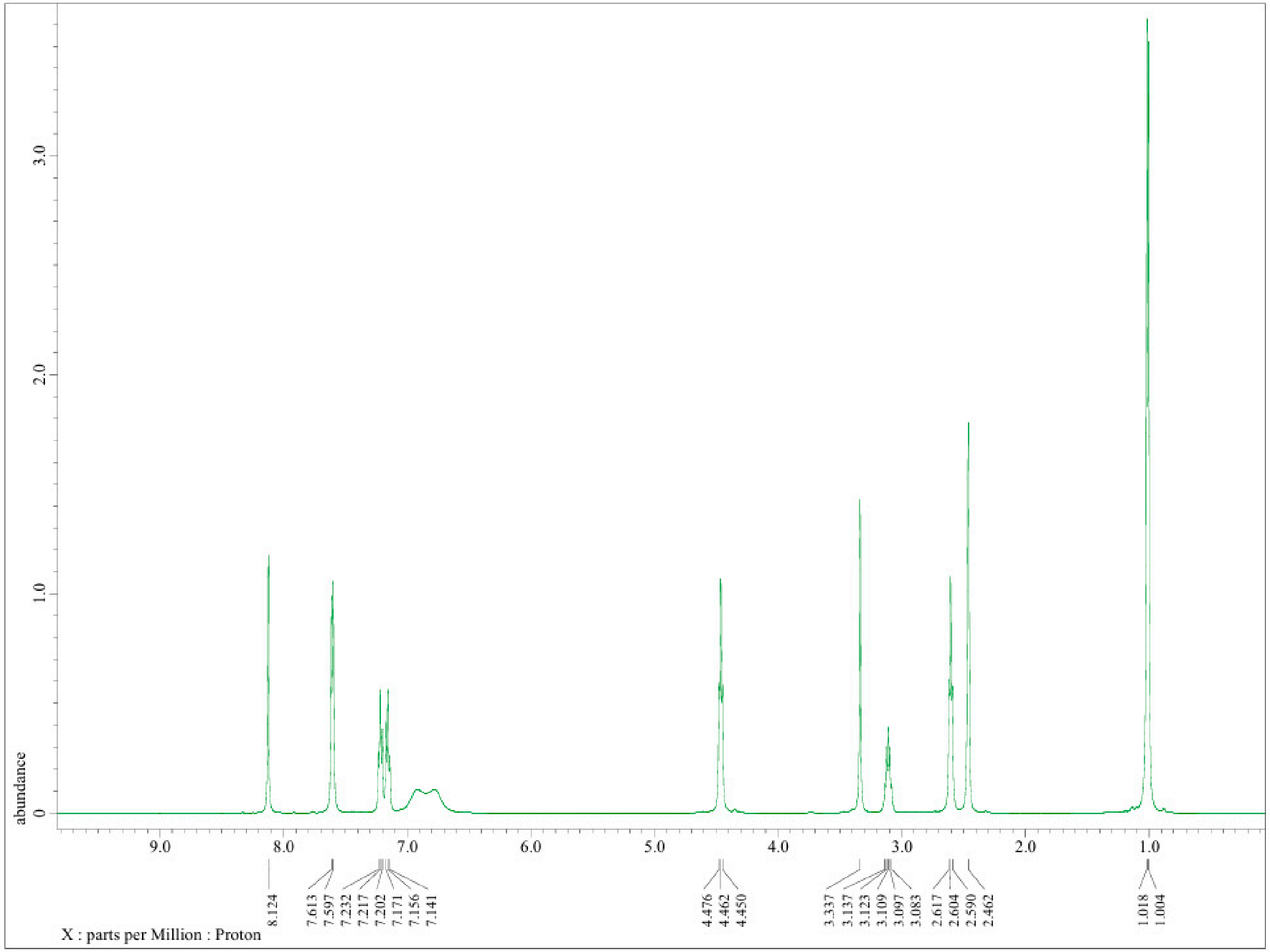

Figure S17. ^{1}H NMR spectrum of O-isopropylsulfonyl-β-(benzimidazole-1-yl)propioamidoxime hydrochloride (**4**).

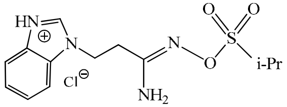

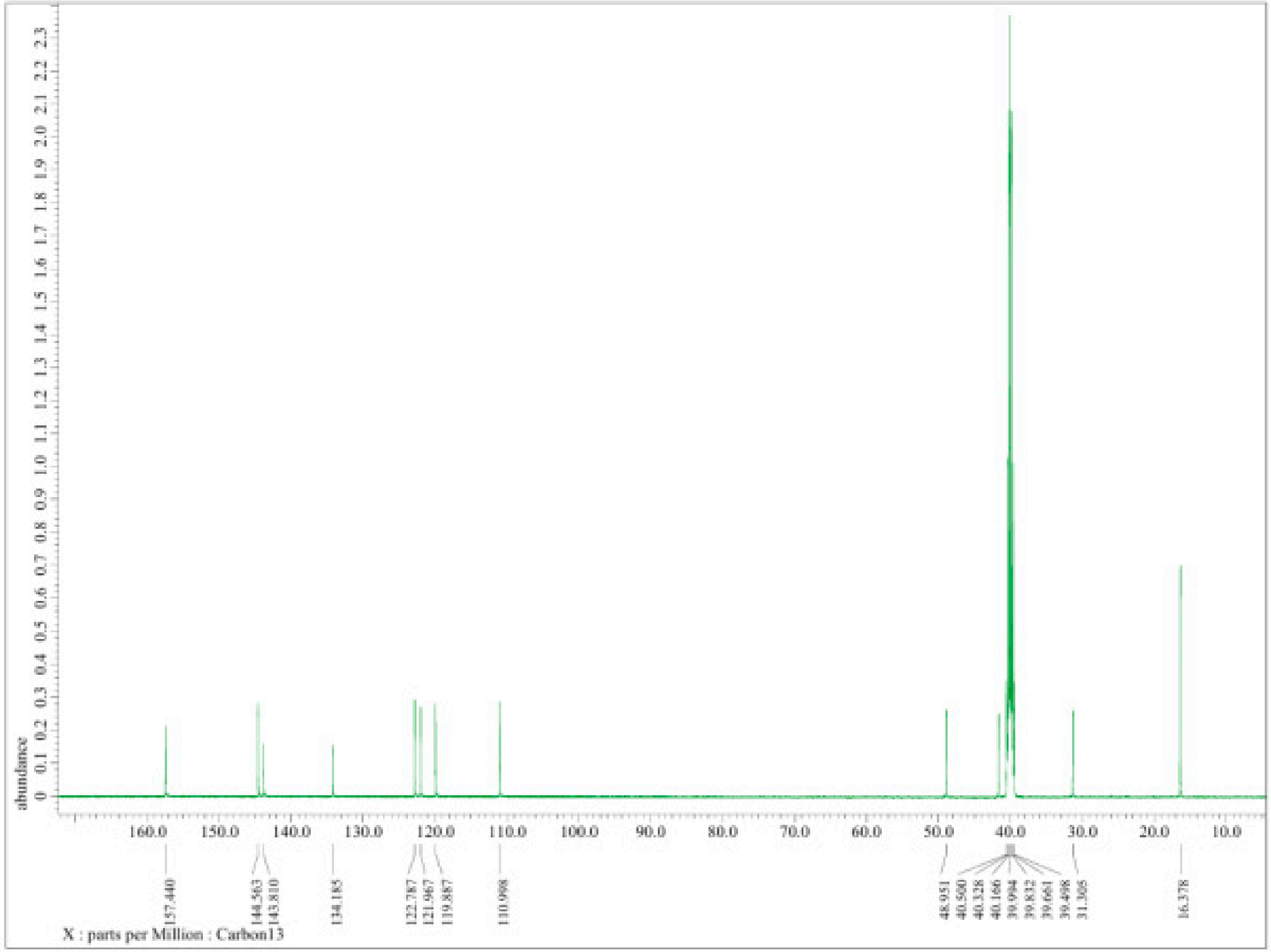

Figure S18. ^{13}C NMR spectrum of O-isopropylsulfonyl-β-(benzimidazole-1-yl)propioamidoxime hydrochloride (**4**).

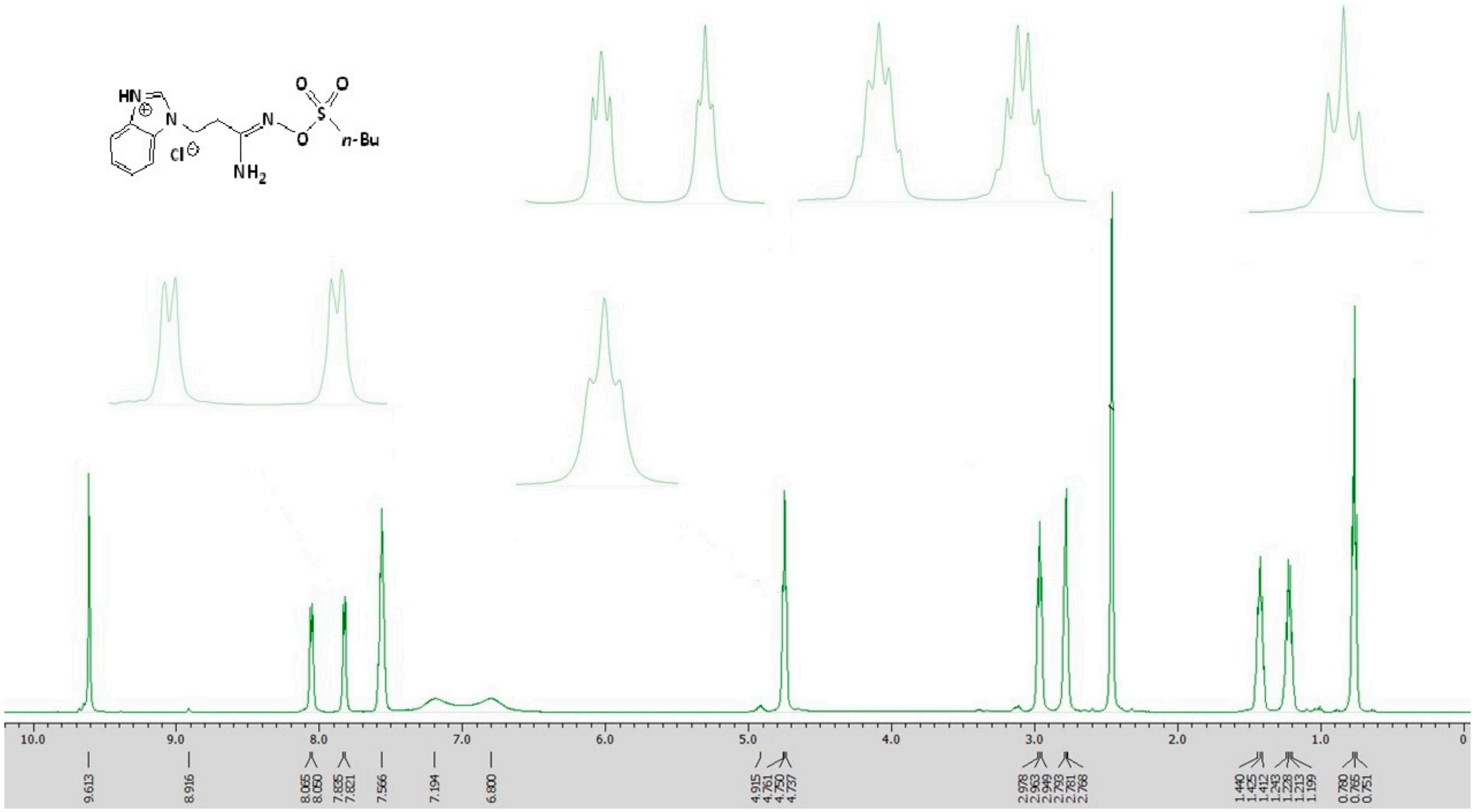

Figure S19. ^{1}H NMR spectrumof O-*n*-butylsulfonyl-β-(benzimidazole-1-yl)propioamidoxime hydrochloride (**5**).

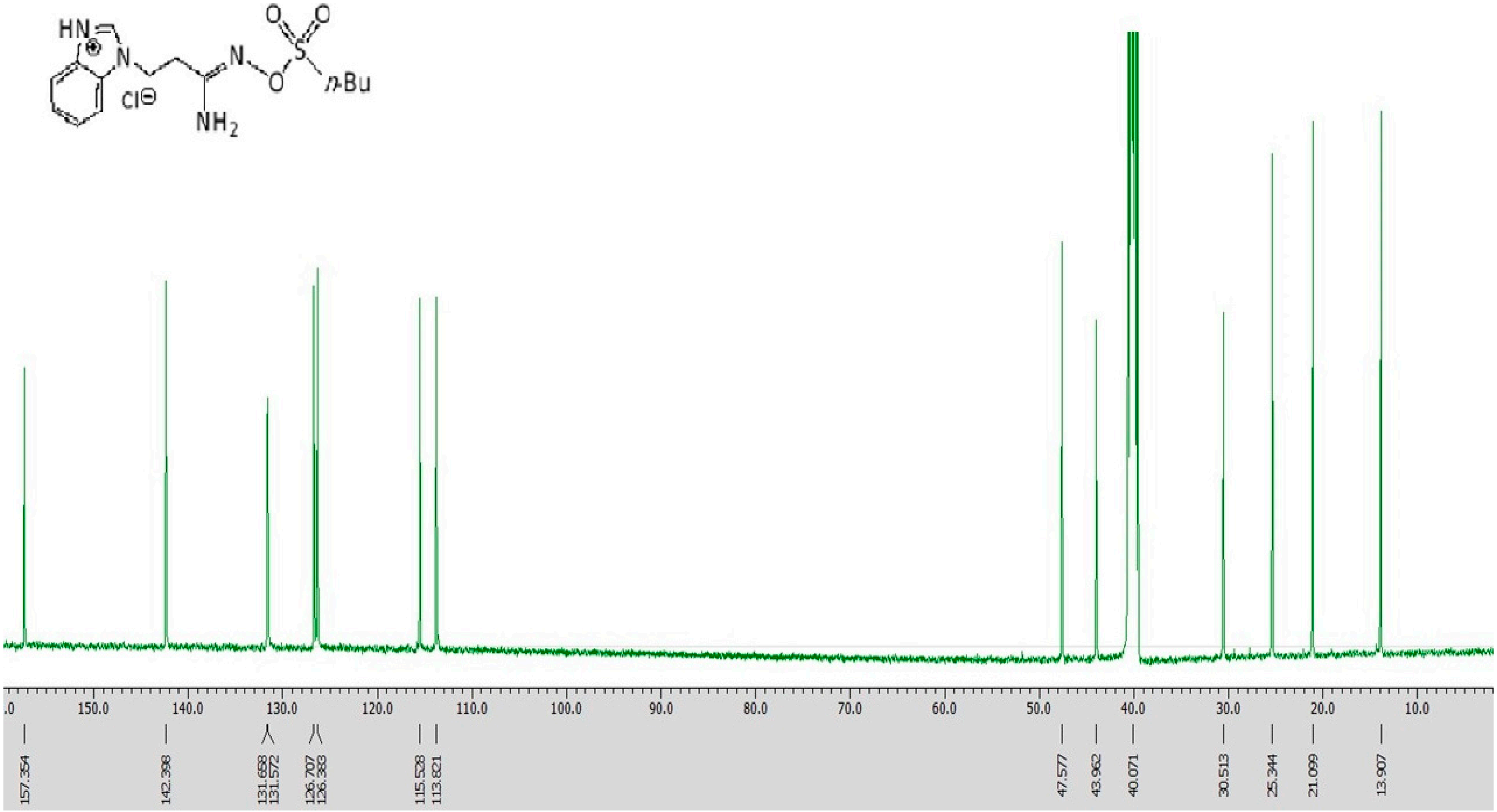

Figure S20. ^{13}C NMR spectrum of O-*n*-butylsulfonyl-β-(benzimidazole-1-yl)propioamidoxime hydrochloride (**5**).

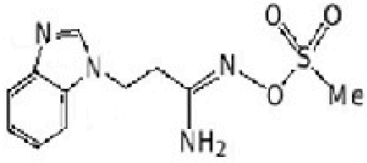

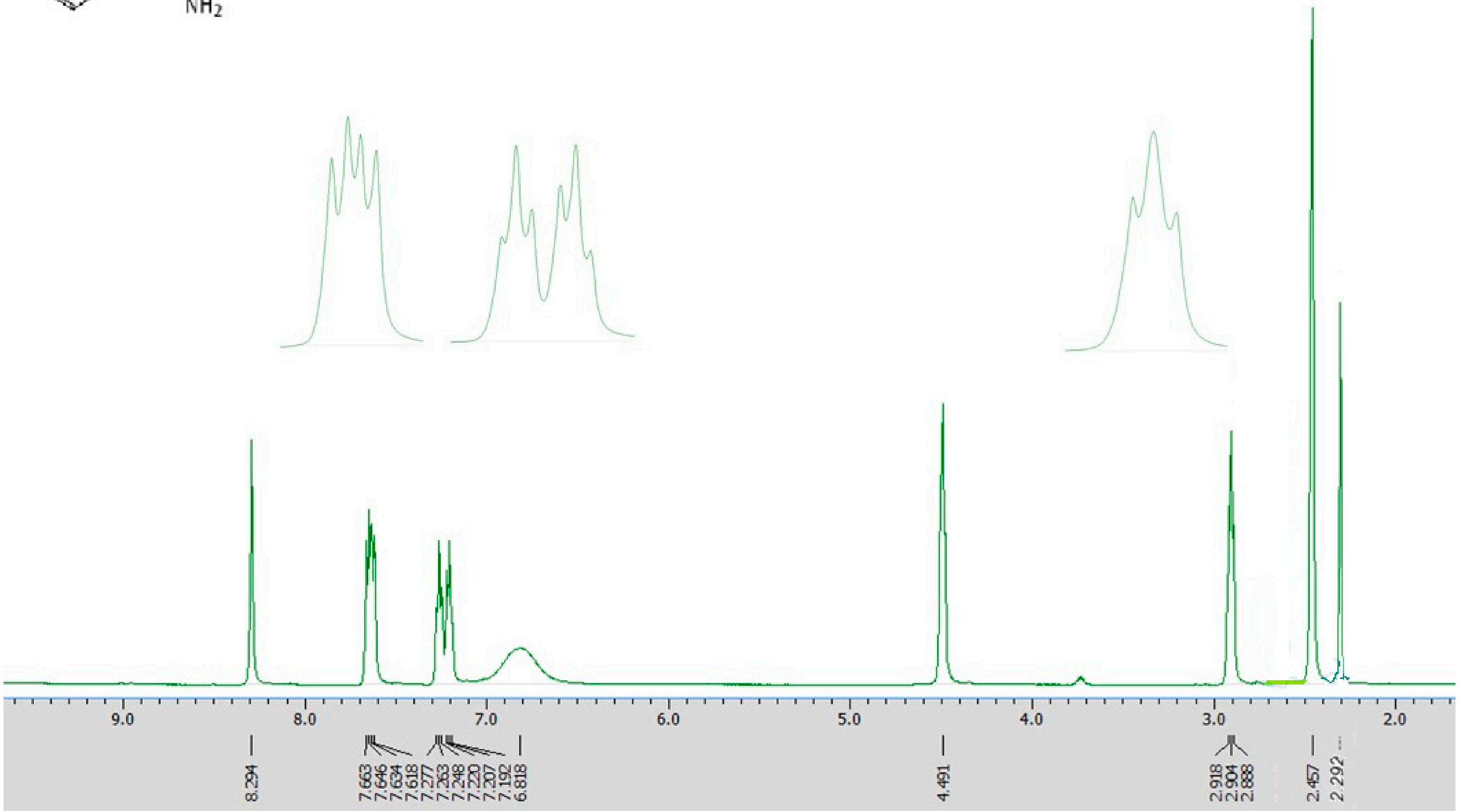

Figure S21. ^{1}H NMR spectrum of O-methylsulfonyl-β-(benzimidazole-1-yl)propioamidoxime (**6**).

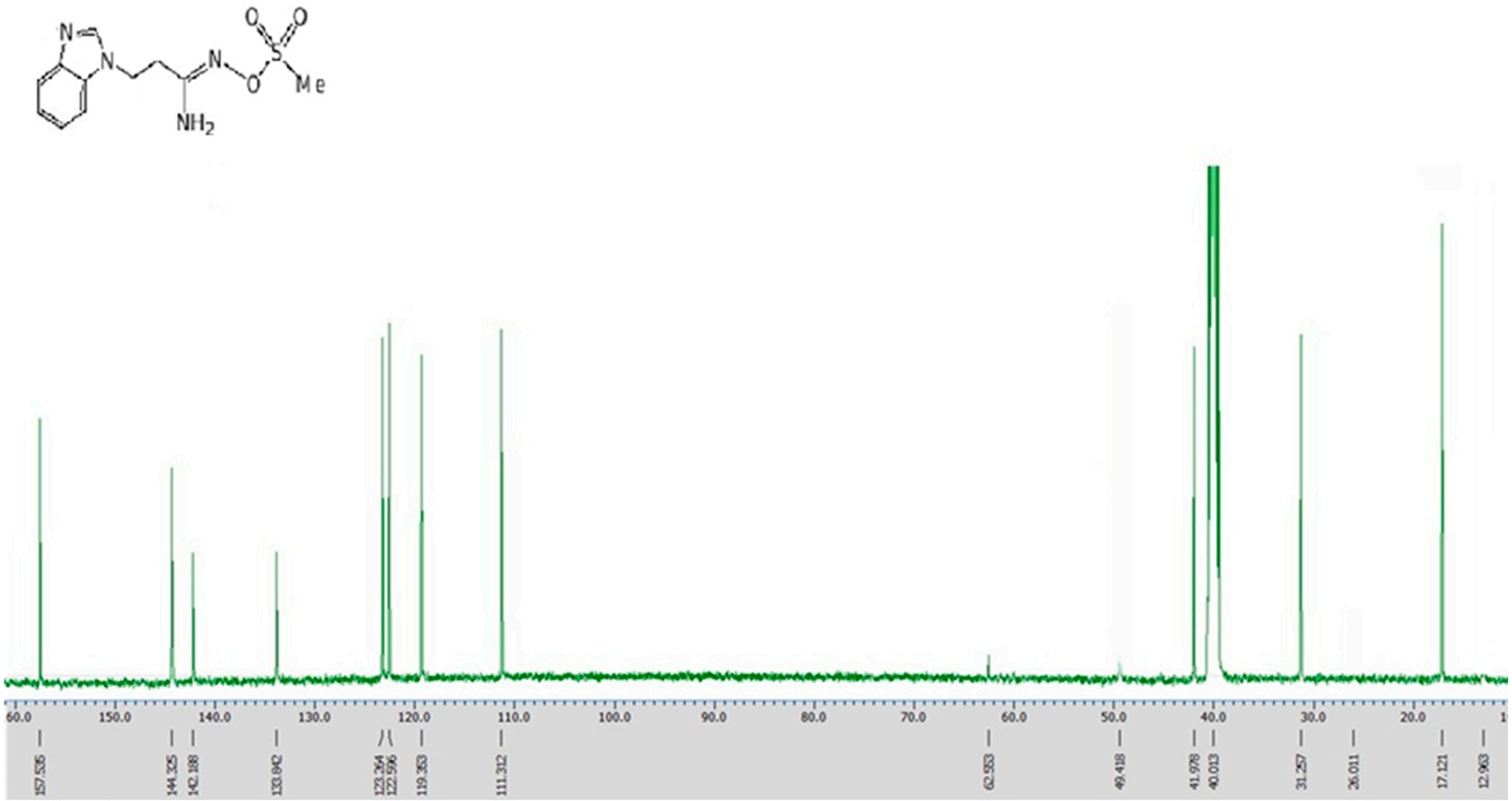

Figure S22. ^{13}C NMR spectrum of O-methylsulfonyl-β-(benzimidazole-1-yl)propioamidoxime (**6**).

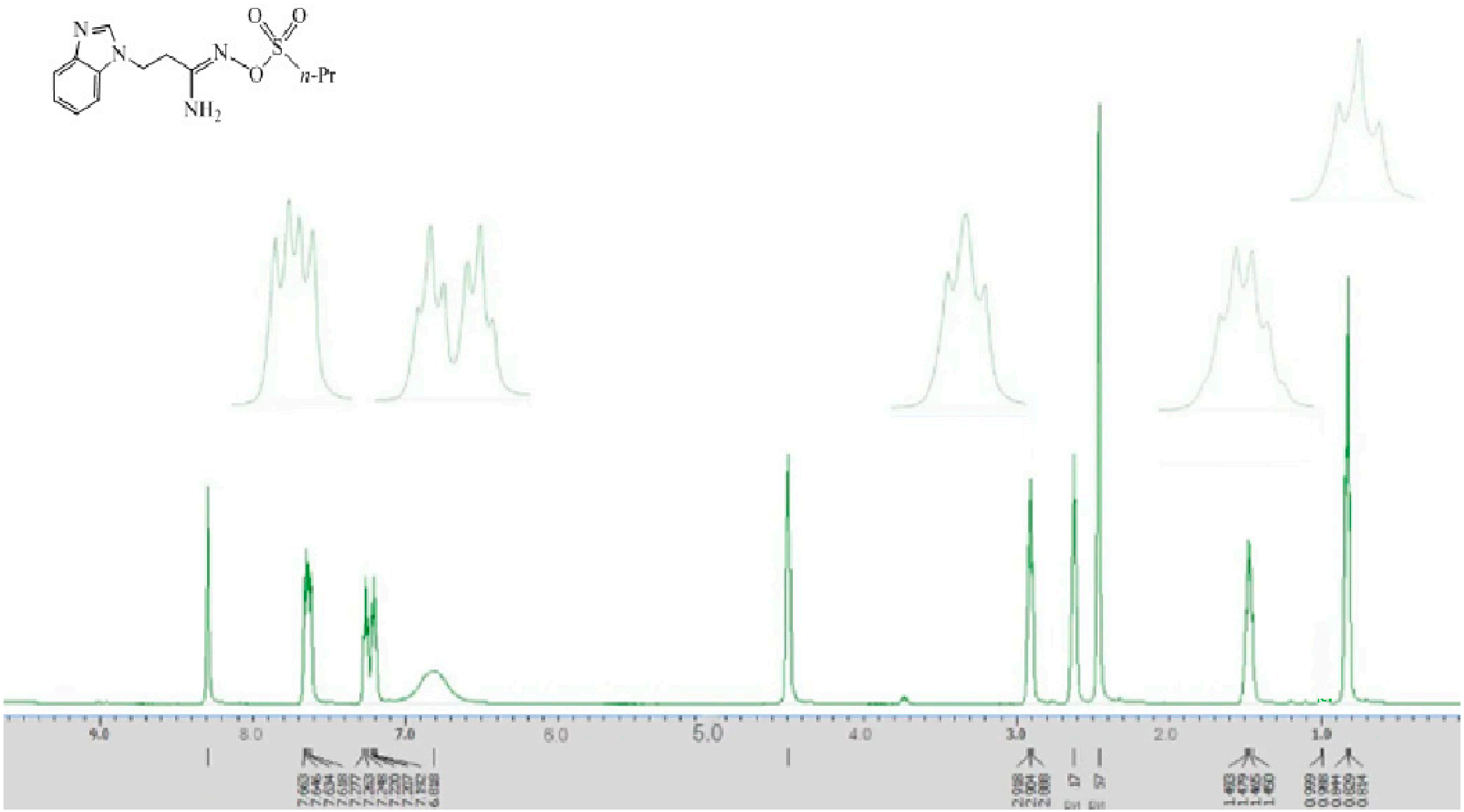

Figure S23. ^{1}H NMR spectrum of O-*n*-propylsulfonyl-β-(benzimidazole-1-yl)propioamidoxime (**7**).

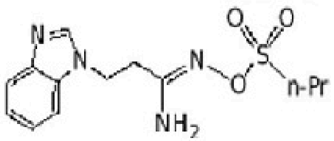

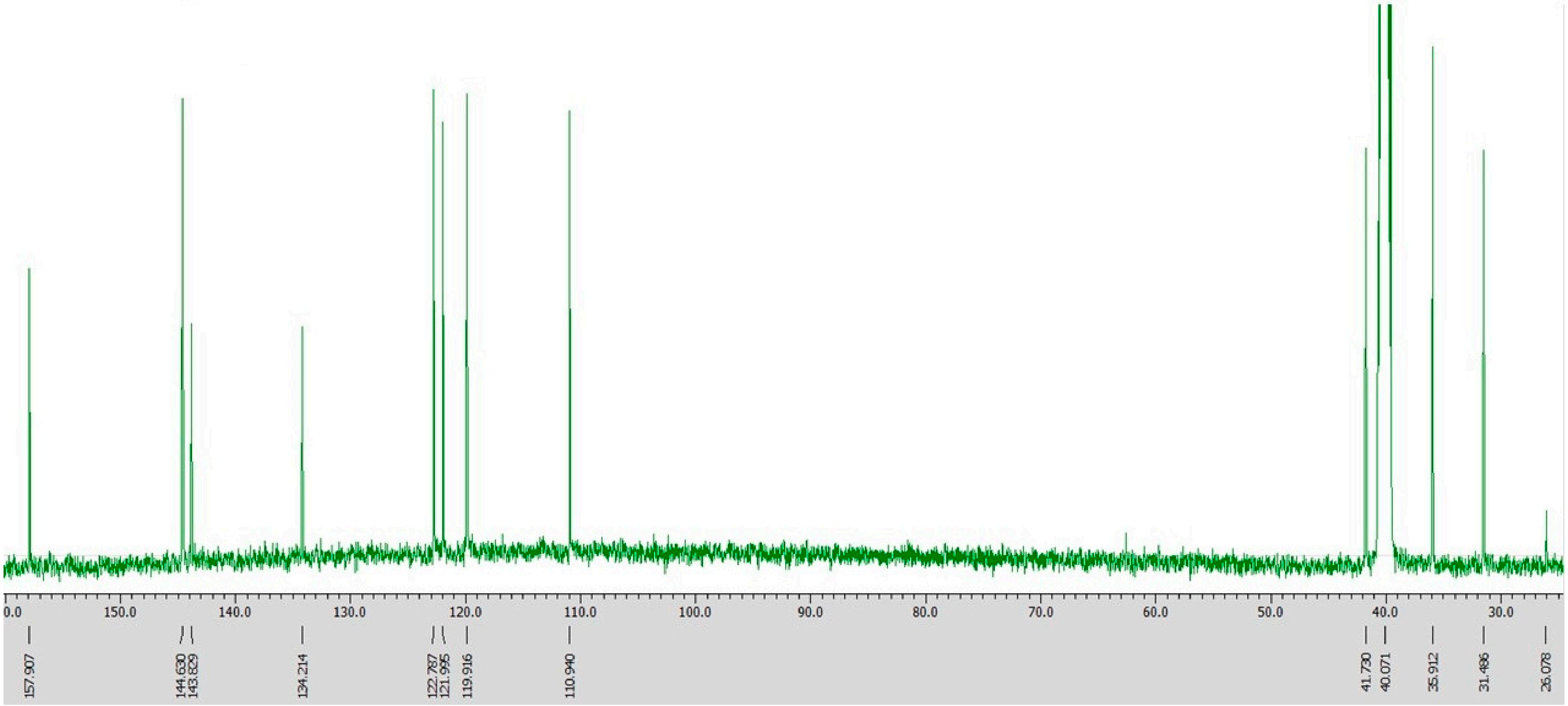

Figure S24. ^{13}C NMR spectrum of O-*n*-propylsulfonyl-β-(benzimidazole-1-yl)propioamidoxime (**7**).

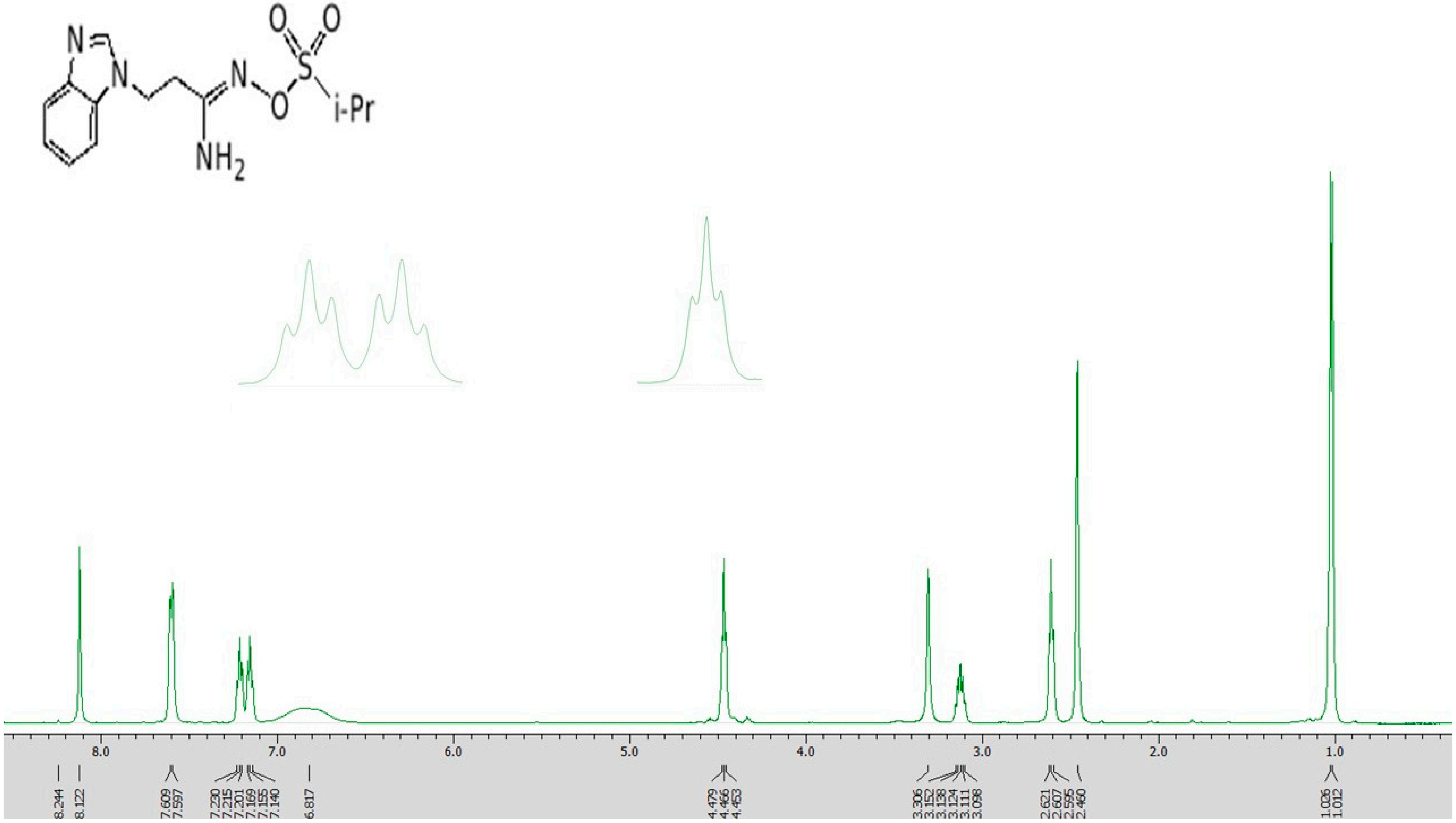

Figure S25. ^{1}H NMR spectrum of O-isopropylsulfonyl-β-(benzimidazole-1-yl)propioamidoxime (**8**).

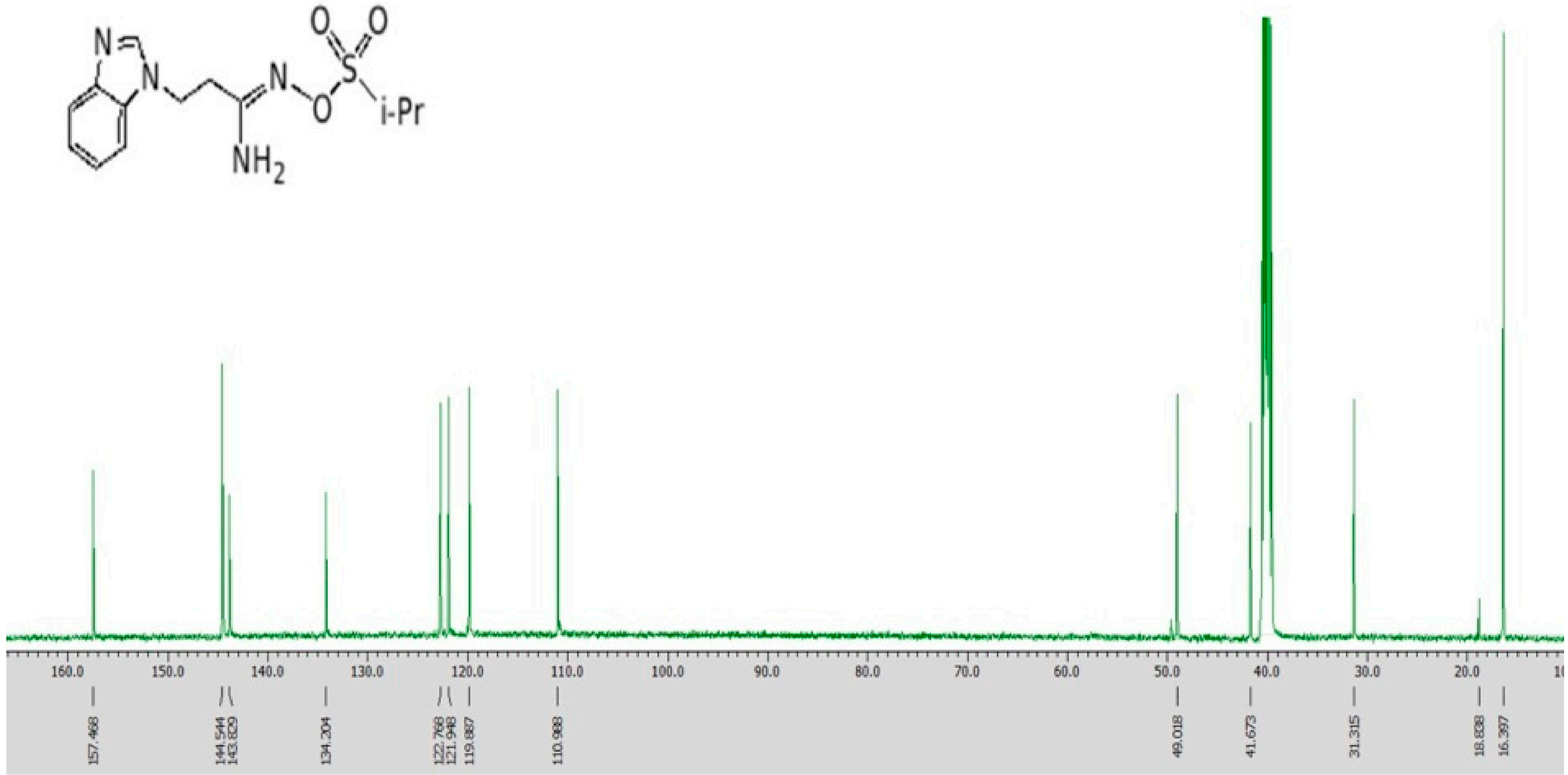

Figure S26. ^{13}C NMR spectrum of O-isopropylsulfonyl-β-(benzimidazole-1-yl)propioamidoxime (**8**).

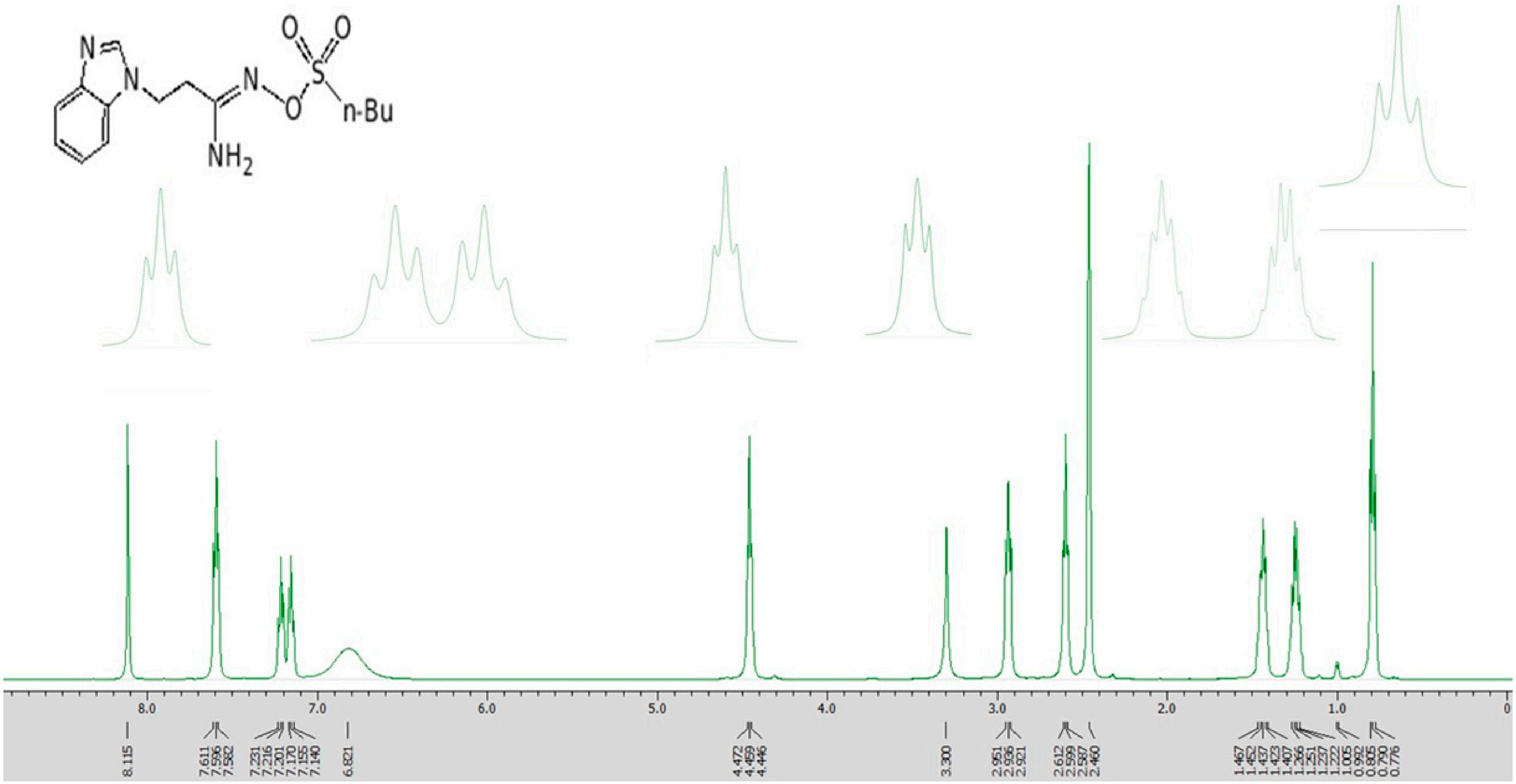

Figure S27. ^{1}H NMR spectrum of O-*n*-butylsulfonyl-β-(benzimidazole-1-yl)propioamidoxime (**9**).

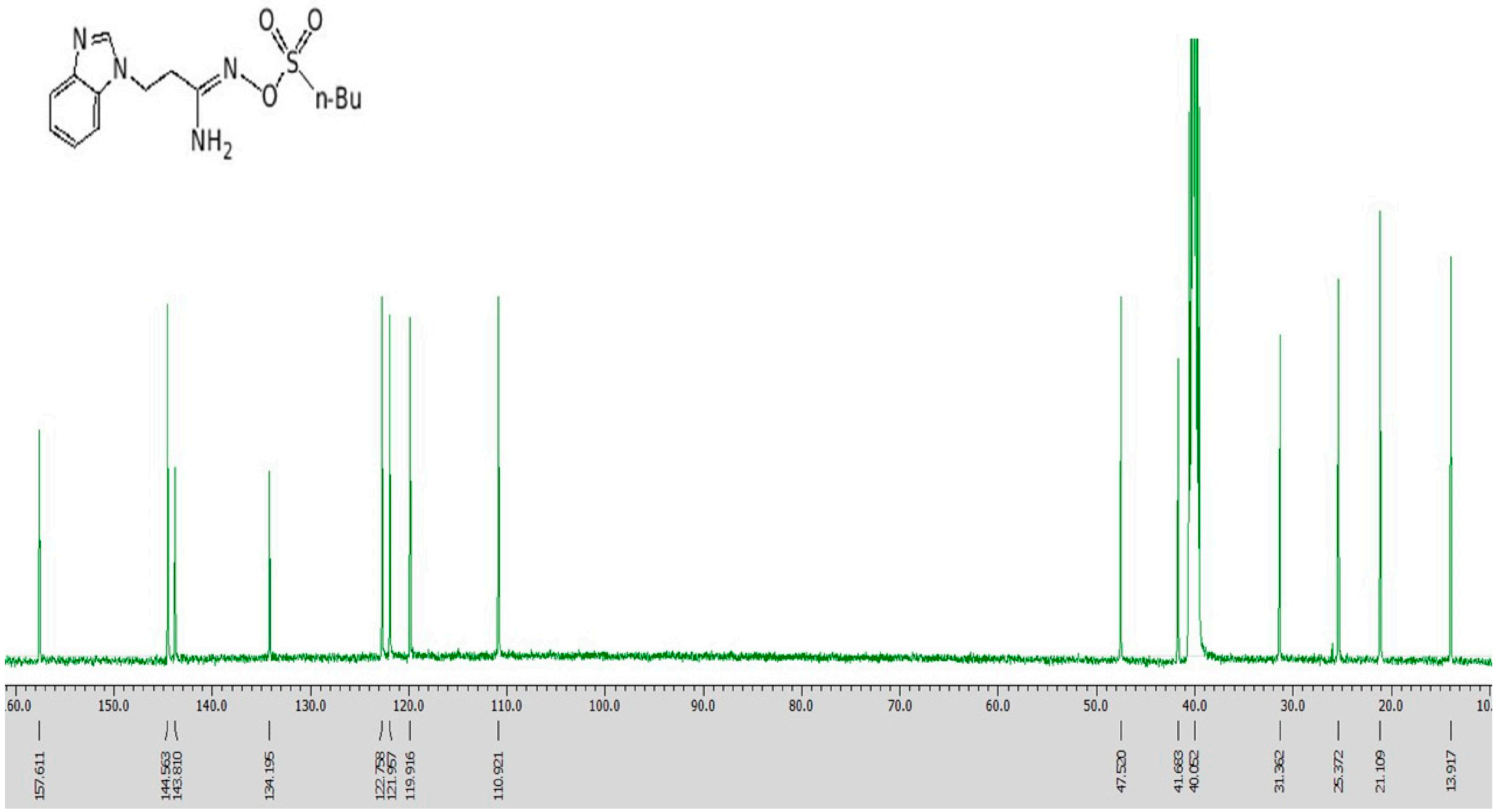

Figure S28. ^{13}C NMR spectrum of O-*n*-butylsulfonyl-β-(benzimidazole-1-yl)propioamidoxime (**9**).

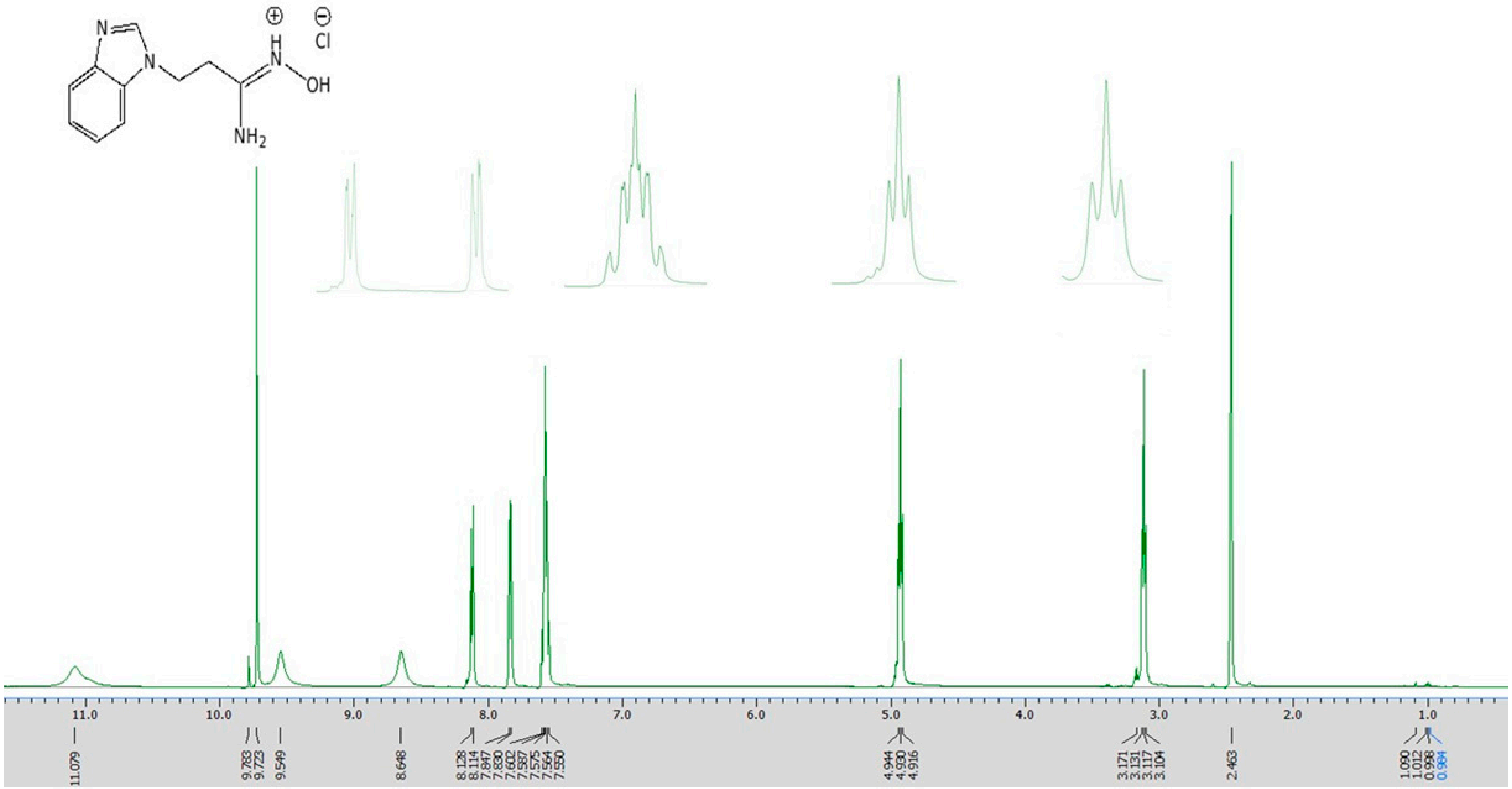

Figure S29. ^{1}H NMR spectrum of β-(benzimidazole-1-yl)propioamidoxime hydrochloride (**10**).

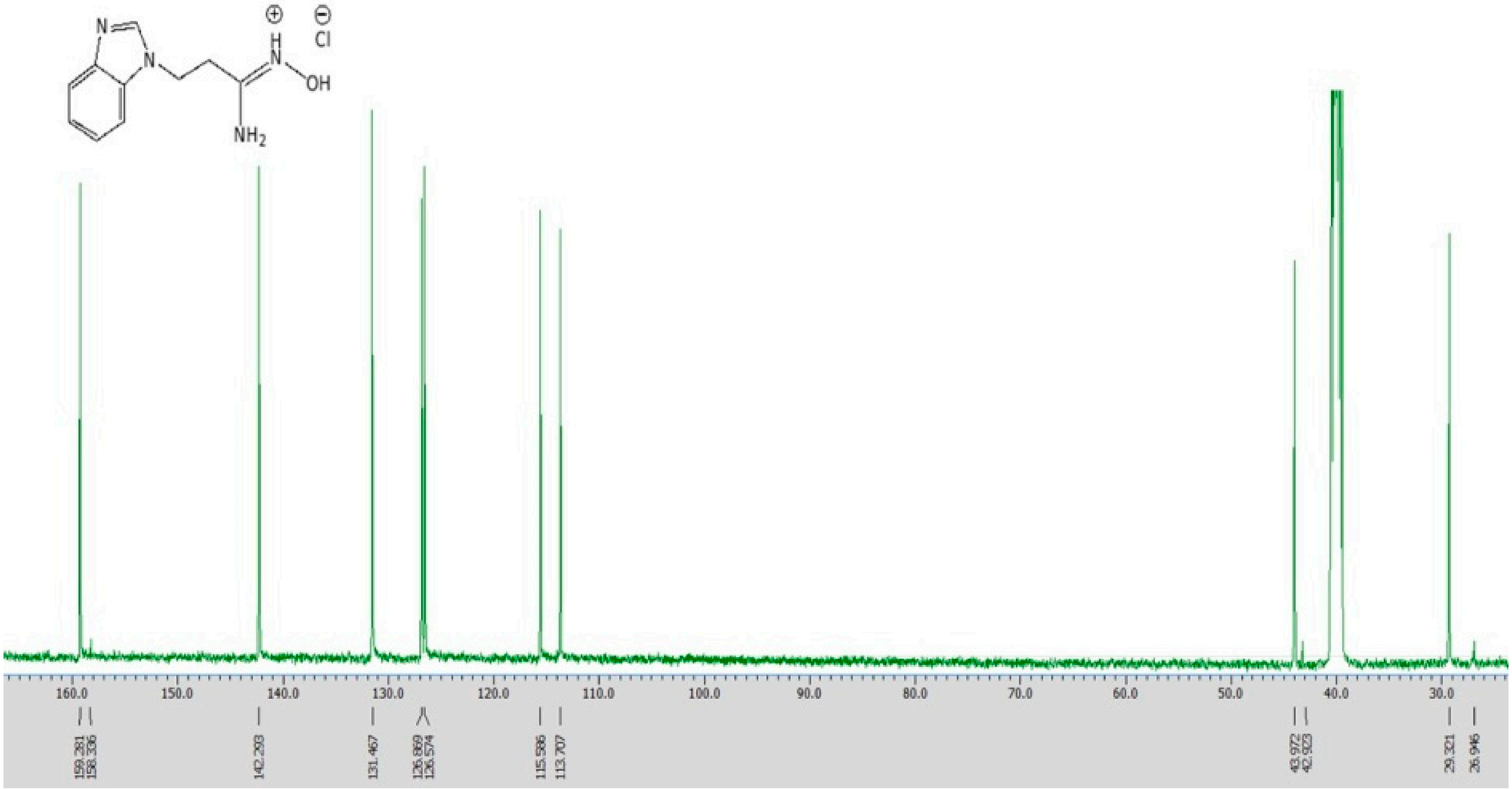

Figure S30. ^{13}C NMR spectrum of β-(benzimidazole-1-yl)propioamidoxime hydrochloride (**10**).

Table S1. Basic crystallographic data of O-alkylsulfonyl-β-(benzimidazole-1-yl)propioamidoximes**3**, **5** and **7**.

Comp	**3** **$(C_{13}H_{19}N_4O_3S)Cl$ $\cdot C_3H_8O$**	**5** **$(C_{14}H_{21}N_4O_{3S})Cl$ $\cdot C_3H_8O$**	**7**
Gross formula	$C_{16}H_{27}ClN_4O_4S$	$C_{17}H_{29}ClN_4O_4S$	$C_{13}H_{18}N_4O_3S$
Molecular weight	406.92	420.95	310.37
T, K	140	140	140
Crystal System	Monoclinic	Monoclinic	Monoclinic
Space Group	$P2_1/c$	$P2_1/c$	$P2_1/c$
Z	4	4	4
a, Å	8.5406(5)	8.5879(5)	10.7338(7)
b, Å	8.1749(5)	8.3129(5)	8.1494(5)
c, Å	29.4074(17)	29.3709(18)	17.3782(11)
b, °	90.208(2)	90.685(2)	96.731(2)
V, Å3	2053.2(2)	2096.6(2)	1509.66(17)
d_{calc}. g·cm^{-3}	1.316	1.334	1.366
m, см-1	0.315	0.311	0.230
F(000)	864	896	656
Number of measured reflections	24745	25328	12060
Number of independent reflections	5577	5719	4054
Number of parameters	273	267	199
R1	0.0389	0.0363	0.0355
wR2	0.0965	0.0940	0.0928
GOF	1.063	1.019	1.006
Residual electron density e×Å-3(dmin/dmax)	0.32/-0.33	0.41/-0.37	0.31/-0.45

Table S2. Basic crystallographic data of β-(benzimidazol-1-yl)propioamide oxime (**1**) and its hydrochloride (**10**).

Comp	**1**	**10**
Gross formula	$C_{10}H_{12}N_4O$	$C_{10}H_{13}ClN_4O$
Molecular mass	204.24	240.69
T. K	100	100
Crystal system	Orthorhombic	Orthorhombic
Space group	$P2_12_12_1$	$Pna2_1$
Z	4	4
a. Å	5.1133(5)	13.450(7)
b. Å	12.526(2)	11.562(5)
c. Å	15.3316(16)	7.333(3)
β. °	90	90
V. $Å^3$	982.0(2)	1140.3(9)
$d_{calc.}$ g•cm^{-3}	1.381	1.402
μ. cm^{-1}	0.095	0.320
F(000)	432	504
Number of measured reflections	8444	5921
Number of independent reflections	2514	1955
Number of parameters	138	152
R1	0.0443	0.0980
wR2	0.0952	0.2111
GOF	1.077	1.042
Residual electron density, e•$Å^{-3}$(d_{min}/d_{max})	0.26/-0.21	0.62/-0.45

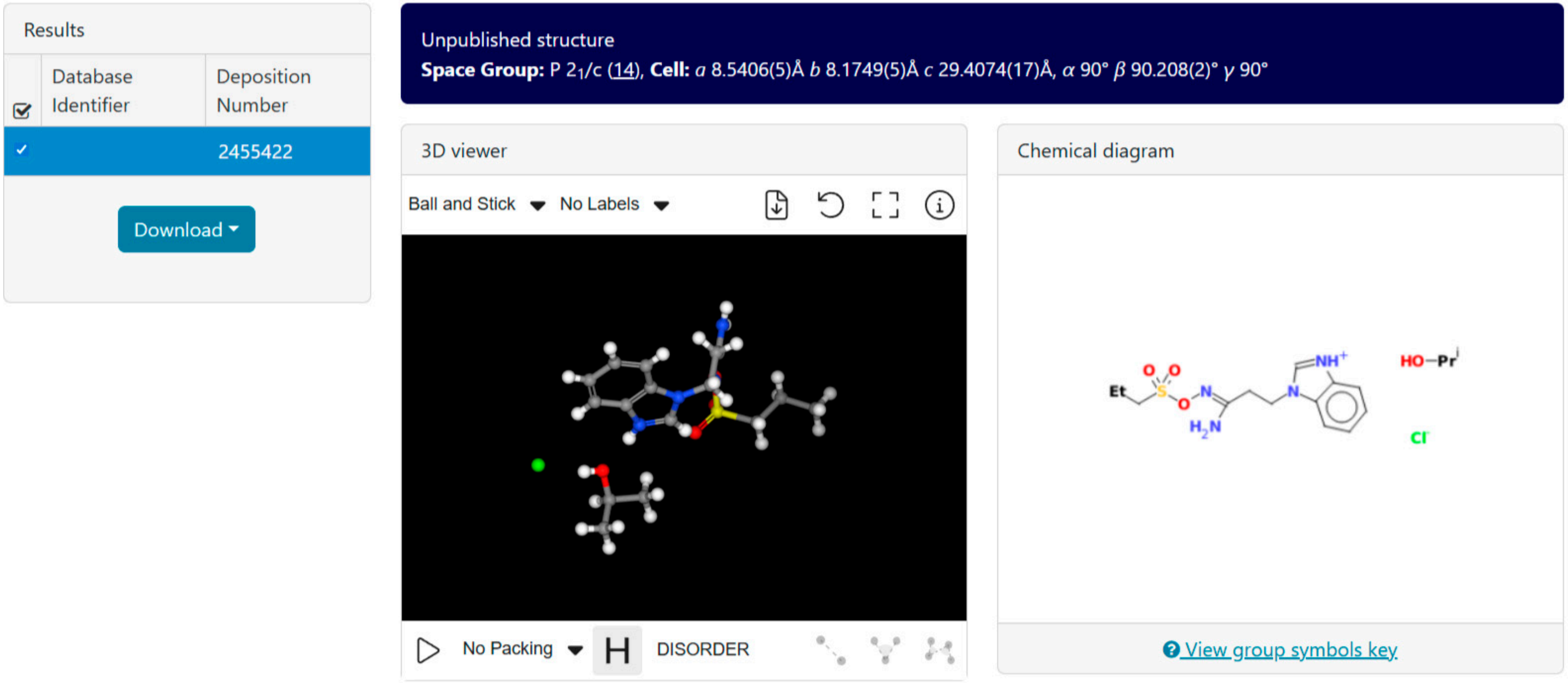

Figure S31. X-Ray structure of O-*n*-propylsulfonyl-β-(benzimidazole-1-yl)propioamidoxime hydrochloride (**3**). URL: http://www.ccdc.cam.ac.uk/services/structures?access=referee&searchdepnums=2455422&searchauthor=Kayukova

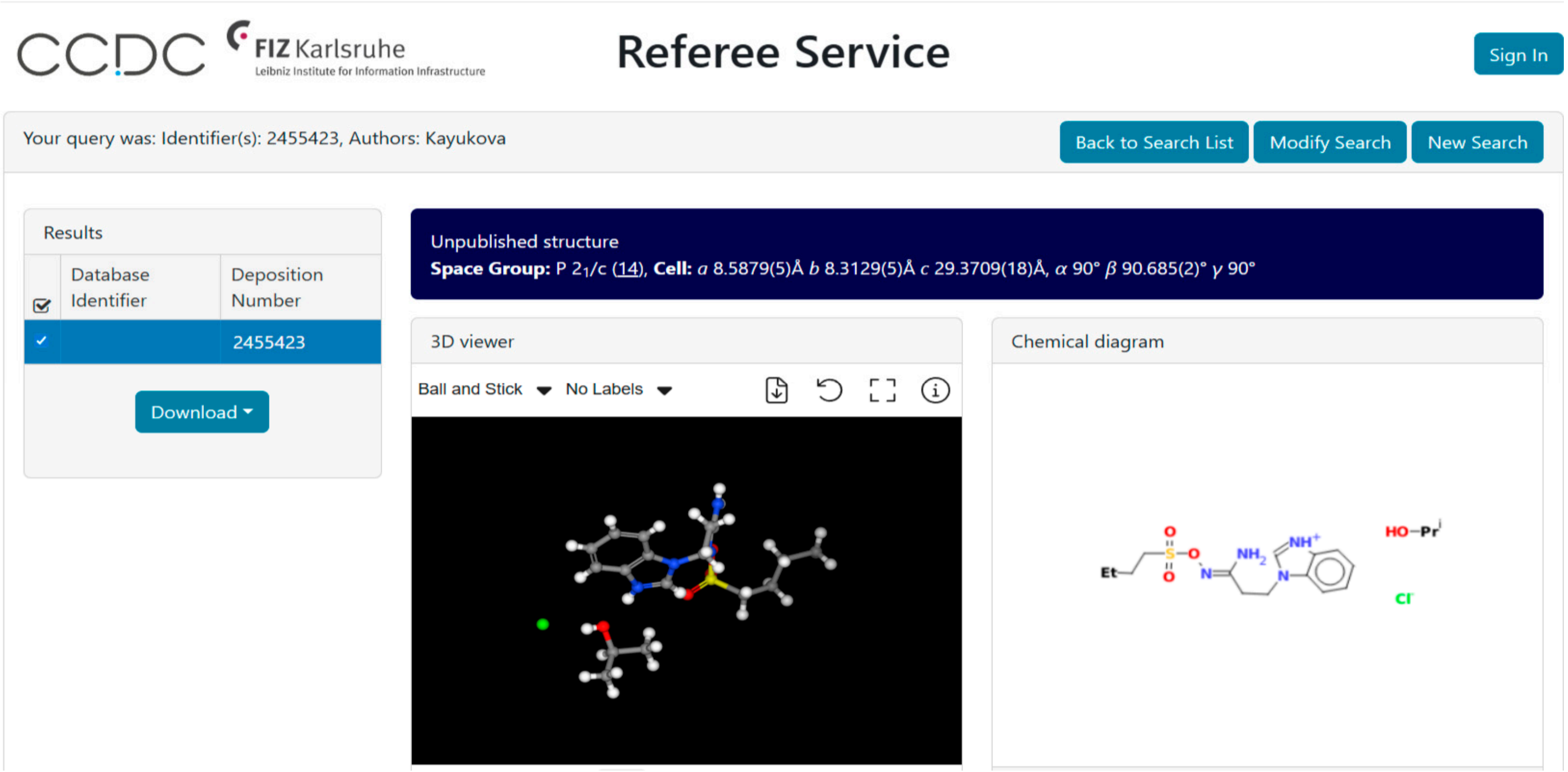

Figure S32. X-Ray structure of O-*n*-butylsulfonyl-β-(benzimidazole-1-yl)propioamidoxime hydrochloride (**5**).
URL: http://www.ccdc.cam.ac.uk/services/structures?access=referee&searchdepnums=2455423&searchauthor=Kayukova

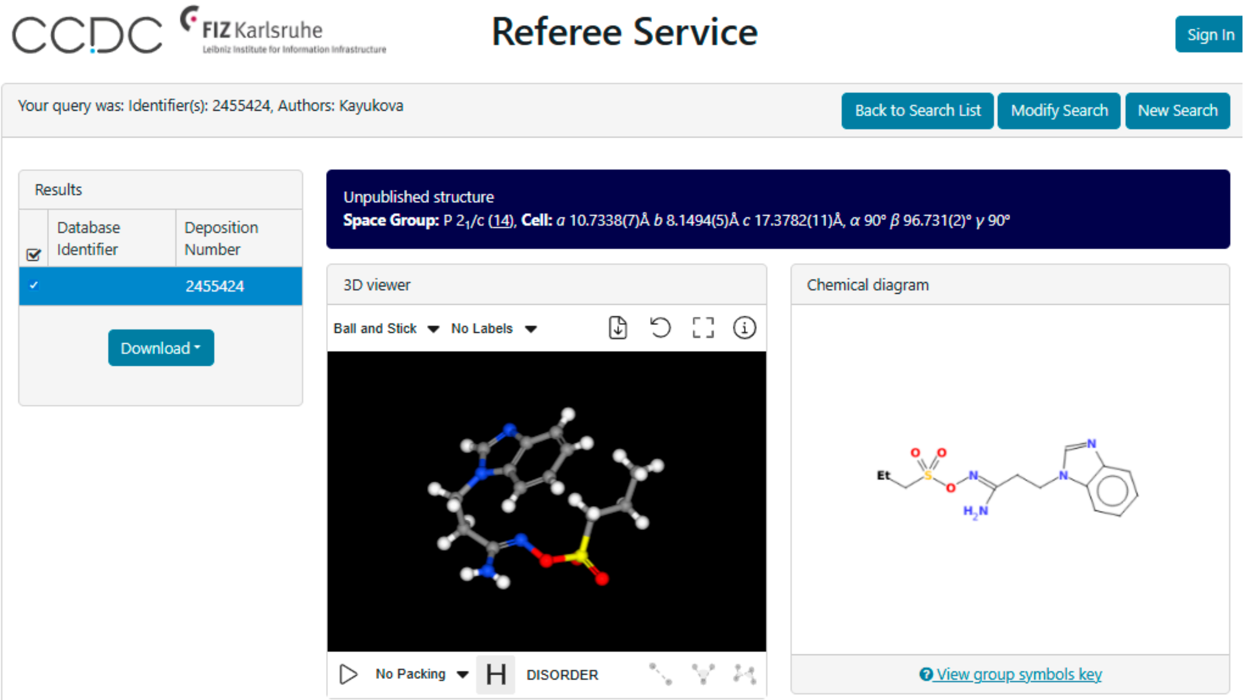

Figure S33. X-Ray structure of O-*n*-propylsulfonyl-β-(benzimidazole-1-yl)propioamidoxime (**7**).
URL: http://www.ccdc.cam.ac.uk/services/structures?access=referee&searchdepnums=2455424&searchauthor=Kayukova

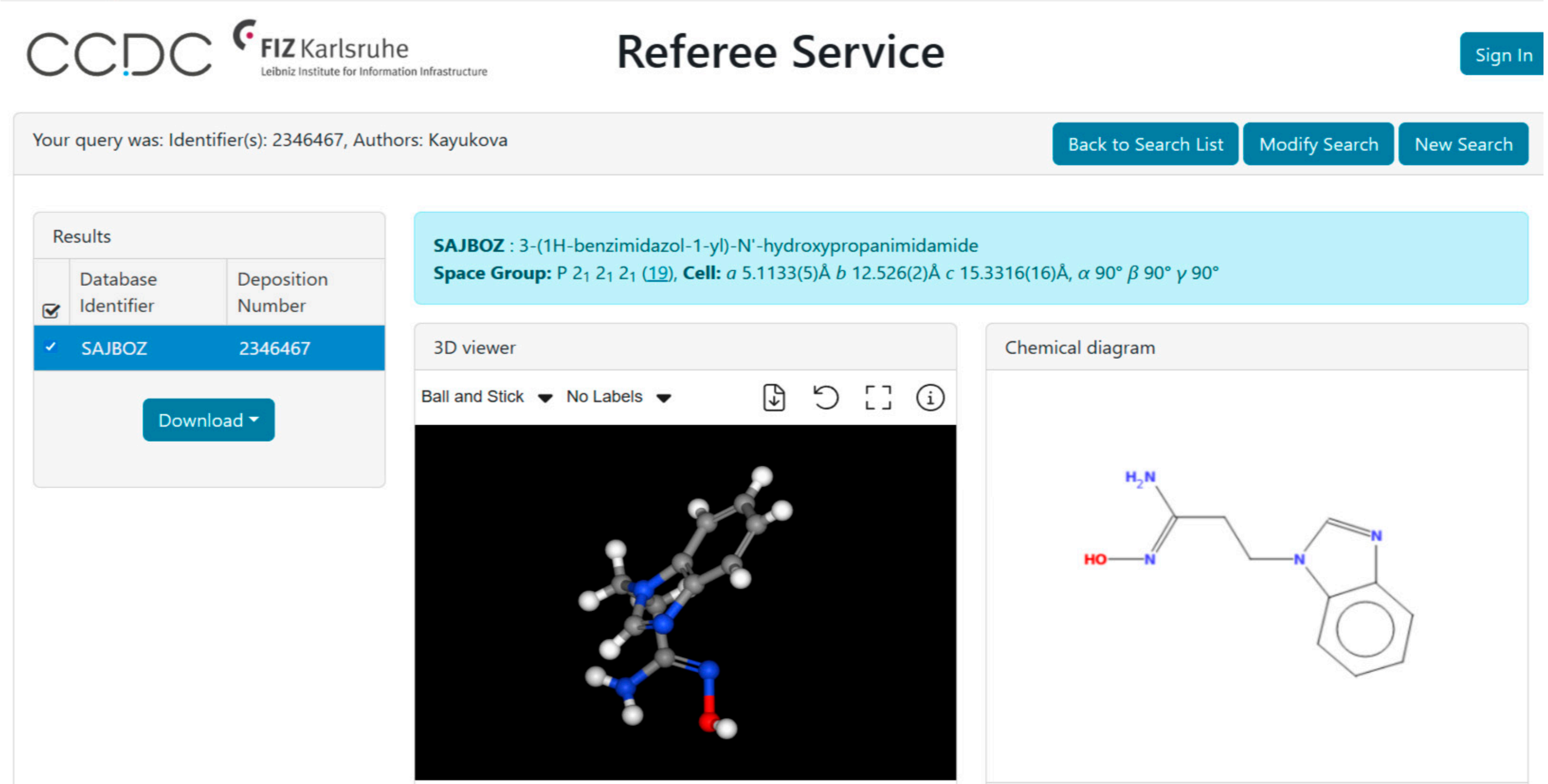

Figure S34. X-Ray structure of of β-(benzimidazole-1-yl)propioamidoxime (**1**).
URL: http://www.ccdc.cam.ac.uk/services/structures?access=referee&searchdepnums=2346467&searchauthor=Kayukova

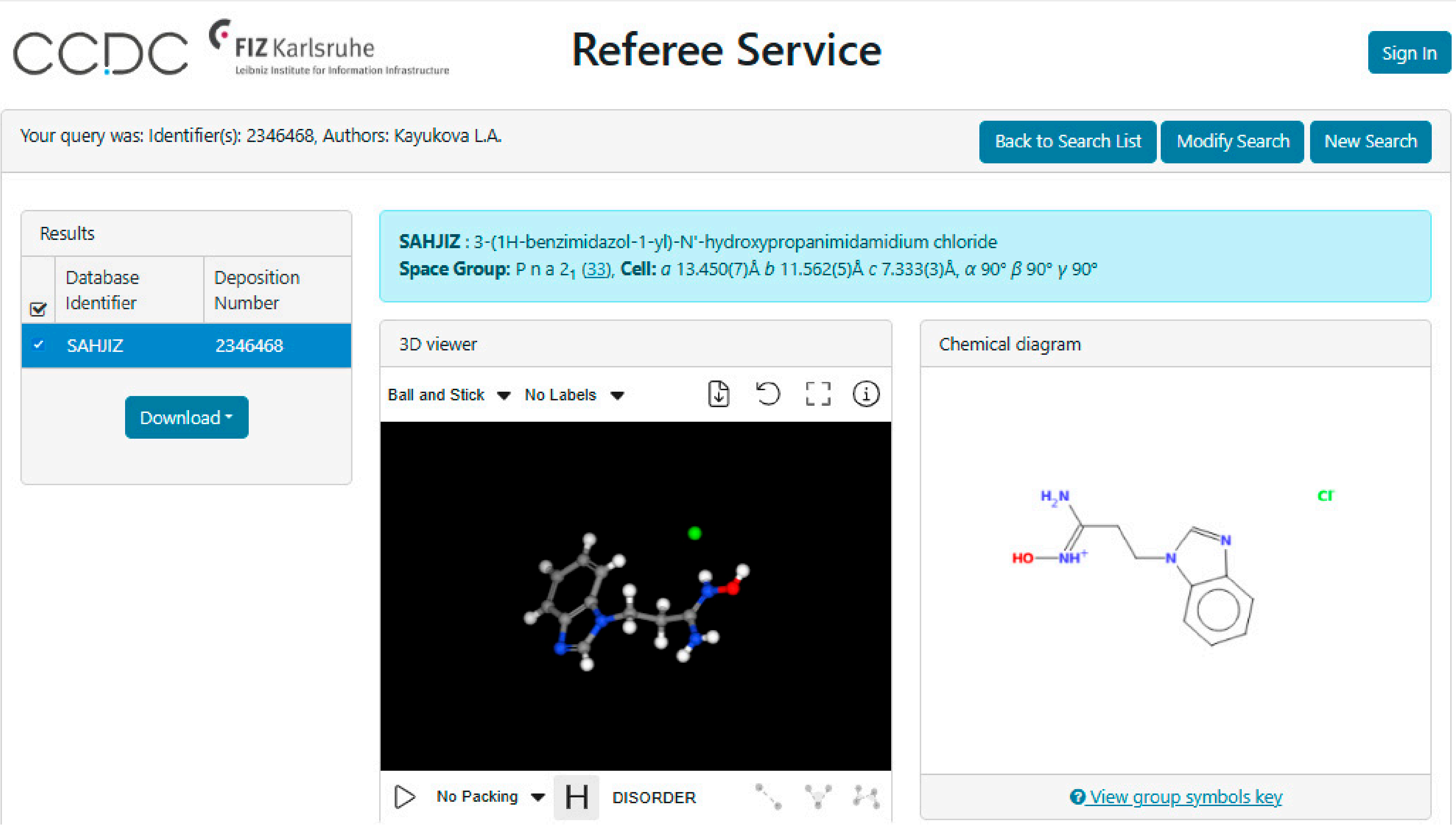

Figure S35. X-Ray structure of of β-(benzimidazole-1-yl)propioamidoxime hydrochloride (**10**).
URL: http://www.ccdc.cam.ac.uk/services/structures?access=referee&searchdepnums=2346468&searchauthor=Kayukova
